# Effect of 8-form Tai Chi vs health education on mobility in older adults with sarcopenic knee osteoarthritis: A randomized clinical trial

**DOI:** 10.1016/j.ocarto.2026.100881

**Published:** 2026-09-23

**Authors:** Guang-Ming Liu, Li-Ping Chai, Ping Huang, Jia-Yu Yang, Wei-Yi Zhan, Chen-Yi Rong, Lin-Fei Yang, Jiao-Jie Zha, Wen-Ting Zhang, Bo Sun, Hong-Sheng Zhan

**Affiliations:** a Xiangshan Hospital of Traditional Chinese Medicine, 528 Fuxing Middle Road, Huangpu District, Shanghai, 200020, China; b Shanghai Institute of Traumatology and Orthopaedics, Ruijin Hospital, Shanghai Jiao Tong University School of Medicine, 197 Ruijin 2nd Road, Shanghai, 200025, China; c Shanghai Huangpu District Yuyuan Community Health Service Center, Lane 1525, Zhonghua Road, Huangpu District, Shanghai, 200021, China; d Shi's Center of Orthopedics and Traumatology, Shuguang Hospital Affiliated to Shanghai University of Traditional Chinese Medicine, 528 Zhangheng Road, Pudong New Area, Shanghai, 201203, China

**Keywords:** Sarcopenic knee osteoarthritis, Tai Chi, Randomized clinical trial, Mobility, Older adults

## Abstract

**Objective:**

To evaluate whether a 12-week simplified 8-form Tai Chi program improves mobility in older adults with sarcopenic knee osteoarthritis (SPKOA) compared with health education plus progressive home exercise.

**Method:**

This assessor-masked, 2-site trial randomized 96 adults aged 65–85 years with SPKOA 1:1 to Tai Chi or health education plus home exercise. The primary outcome was change in Timed Up and Go (TUG) time at week 12. Site-adjusted categorical-time linear mixed-effects models were used. A TUG improvement of at least 2.8 s defined individual response.

**Results:**

At week 12, the adjusted between-group difference in TUG change was −2.52 s (95% CI, −3.43 to −1.61; p < 0.001), favoring Tai Chi but slightly below the prespecified individual improvement threshold. TUG responders were more frequent with Tai Chi (28/48 [58.3%] vs 6/48 [12.5%]; adjusted risk ratio, 4.81 [95% CI, 2.29–10.07]). At week 26, the TUG difference was −1.46 s (95% CI, −2.41 to −0.52). Western Ontario and McMaster Universities Osteoarthritis Index (WOMAC) pain favored Tai Chi, whereas visual analog scale pain showed no clear between-group separation. No intervention-related serious adverse events occurred.

**Conclusion:**

Simplified 8-form Tai Chi improved mobility compared with an active health education and home-exercise program in older adults with SPKOA. The average week-12 TUG advantage was slightly below the prespecified individual improvement threshold, but clinically important TUG responses were more frequent with Tai Chi and a smaller mean benefit persisted at week 26.

**Trial registration:**

International Traditional Medicine Clinical Trial Registry: ITMCTR2025001324.

## 1 Introduction

Sarcopenic knee osteoarthritis (SPKOA) refers to the coexistence of sarcopenia and knee osteoarthritis in older adults. Both conditions are associated with pain, muscle weakness, impaired physical performance, and loss of independence, and their coexistence may further compromise mobility [[1], [2], [3]]. In community-dwelling older adults with SPKOA, potentially relevant clinical factors include knee pain, body mass index, physical activity, calf circumference, and metabolic comorbidity [4]. These observations highlight the need for interventions that address mobility and joint symptoms while remaining feasible for older adults with reduced muscle function [5,6].

Tai Chi combines slow, controlled movement with repeated weight shifting, postural adjustment, balance control, and sustained attention. Randomized trials and systematic reviews in knee osteoarthritis and older populations suggest that Tai Chi may improve physical function, balance, pain, and walking-related performance [[7], [8], [9], [10]]. Such effects may be particularly relevant to SPKOA, in which mobility limitations may reflect not only reduced muscle mass or strength but also impaired postural control, altered movement strategies, and pain-related adaptations [11]. However, most previous Tai Chi studies have enrolled people with knee osteoarthritis or older adults more broadly, and randomized evidence specifically in older adults with confirmed SPKOA remains limited.

We therefore conducted a 2-arm randomized clinical trial comparing a standardized simplified 8-form Tai Chi program with health education plus a progressive home-exercise program in community-dwelling older adults with SPKOA. The primary hypothesis was that Tai Chi would produce greater improvement in Timed Up and Go (TUG) performance from baseline to week 12. Secondary outcomes, prespecified responder analyses, and exploratory subgroup analyses were used to characterize the broader pattern and clinical interpretability of treatment effects.

## 2 Methods

### 2.1 Study design and setting

This 2-arm, parallel-group, assessor-masked superiority randomized clinical trial was conducted at 2 clinical sites in Huangpu District, Shanghai, China: Xiangshan Hospital of Traditional Chinese Medicine and Yuyuan Community Health Service Center. The trial was reported in accordance with the Consolidated Standards of Reporting Trials (CONSORT) 2025 statement [12]. The Ethics Committee of Xiangshan Hospital of Traditional Chinese Medicine approved the protocol (XSEC2025018) on January 30, 2025, and all participants provided written informed consent.

Trial registration was submitted to the International Traditional Medicine Clinical Trial Registry before enrollment and publicly posted on July 2, 2025 after administrative processing (ITMCTR2025001324; http://itmctr.ccebtcm.org.cn/). Recruitment began on February 1, 2025. The protocol and statistical analysis plan (SAP) are provided in Supplements 2 and 3; SAP version 1.2 (December 20, 2024) was finalized before recruitment and outcome analysis.

### 2.2 Participants

Community-dwelling adults aged 65–85 years were recruited from outpatient services at both sites. SPKOA was defined as sarcopenia plus radiographic knee osteoarthritis. Sarcopenia followed the 2019 Asian Working Group for Sarcopenia criteria: low muscle strength (handgrip strength <28 kg for men or <18 kg for women) plus low physical performance (usual gait speed ≤1.0 m/s over 6 m or TUG >20 s) or low skeletal muscle mass (appendicular skeletal muscle mass index [ASMI] <7.0 kg/m^2^ for men or <5.7 kg/m^2^ for women by bioelectrical impedance analysis) [13]. Radiographic knee osteoarthritis was defined as Kellgren-Lawrence (KL) grade I–IV on weight-bearing anteroposterior radiographs [14]. Participants had to be able to walk at least 3 m without an assistive device and provide informed consent.

Major exclusions were inflammatory or secondary arthritis, neurologic disease affecting mobility, previous knee arthroplasty or osteotomy, severe uncontrolled cardiopulmonary or systemic disease, contraindications to moderate-intensity exercise, implanted electronic devices incompatible with bioelectrical impedance analysis, or inability to complete assessments.

### 2.3 Randomization and masking

After baseline assessment, participants were randomized 1:1 to the Tai Chi group (TCG) or health education group (HEG), stratified by site using permuted blocks of randomly varying size (2–6) generated by an independent statistician. Allocation was concealed in sequentially numbered, opaque, sealed envelopes opened after baseline assessment. Participants and instructors were unmasked; outcome assessors and data analysts were masked during assessment and primary analysis.

### 2.4 Interventions

Both programs lasted 12 weeks. The TCG received standardized 8-form Yang-style Tai Chi. Weeks 1–2 comprised 4 supervised 60-min sessions (2 sessions/week) emphasizing movement decomposition, alignment, controlled weight shifting, and breath-movement coordination. During weeks 3–12, home Tai Chi was prescribed 5 days/week for approximately 30 min/day, progressing toward balance-focused practice. The prescribed intensity was Borg CR10 3–5. Participants stopped exercise for concerning symptoms or knee pain >3/10.

The HEG attended 6 structured 60-min health education sessions covering SPKOA, dietary protein, fall prevention, and self-management. No home exercise was prescribed during weeks 1–2. During weeks 3–12, participants performed progressive home exercise 5 days/week for approximately 20 min/day, including strengthening, functional mobility, balance, stair practice, and cognitive-motor tasks; exercise stopped if knee pain exceeded 3/10. Detailed schedules, progression, and safety procedures for both groups are provided in Supplement 1,eAppendix.

### 2.5 Adherence and fidelity

Adherence was monitored weekly from attendance records and WeChat home-practice logs. Supervised adherence was sessions attended divided by sessions prescribed; home adherence was completed practice days divided by 50 prescribed days. The proportion achieving at least 80% adherence was summarized separately for supervised sessions and home practice; session duration and perceived exertion were not part of the adherence definition. Fidelity was supported by standardized materials, instructor checks, and periodic review of participant-submitted practice videos.

### 2.6 Outcome assessments

Masked assessors conducted in-person assessments at baseline and weeks 4, 8, 12, and 26; ASMI was assessed at baseline and week 12 only. The primary outcome was change from baseline to week 12 in TUG time, measured as the time to rise from a standard chair, walk 3 m, turn, return, and sit [15]. Lower values indicate better mobility. A within-participant TUG reduction of 2.82 s has been reported as a minimal clinically important difference in knee osteoarthritis, and an improvement of at least 2.8 s was prespecified as the threshold for clinically important individual improvement [16]. The assessment schedule is summarized in Supplement 1, eFig. 1.

**Fig. 1:**
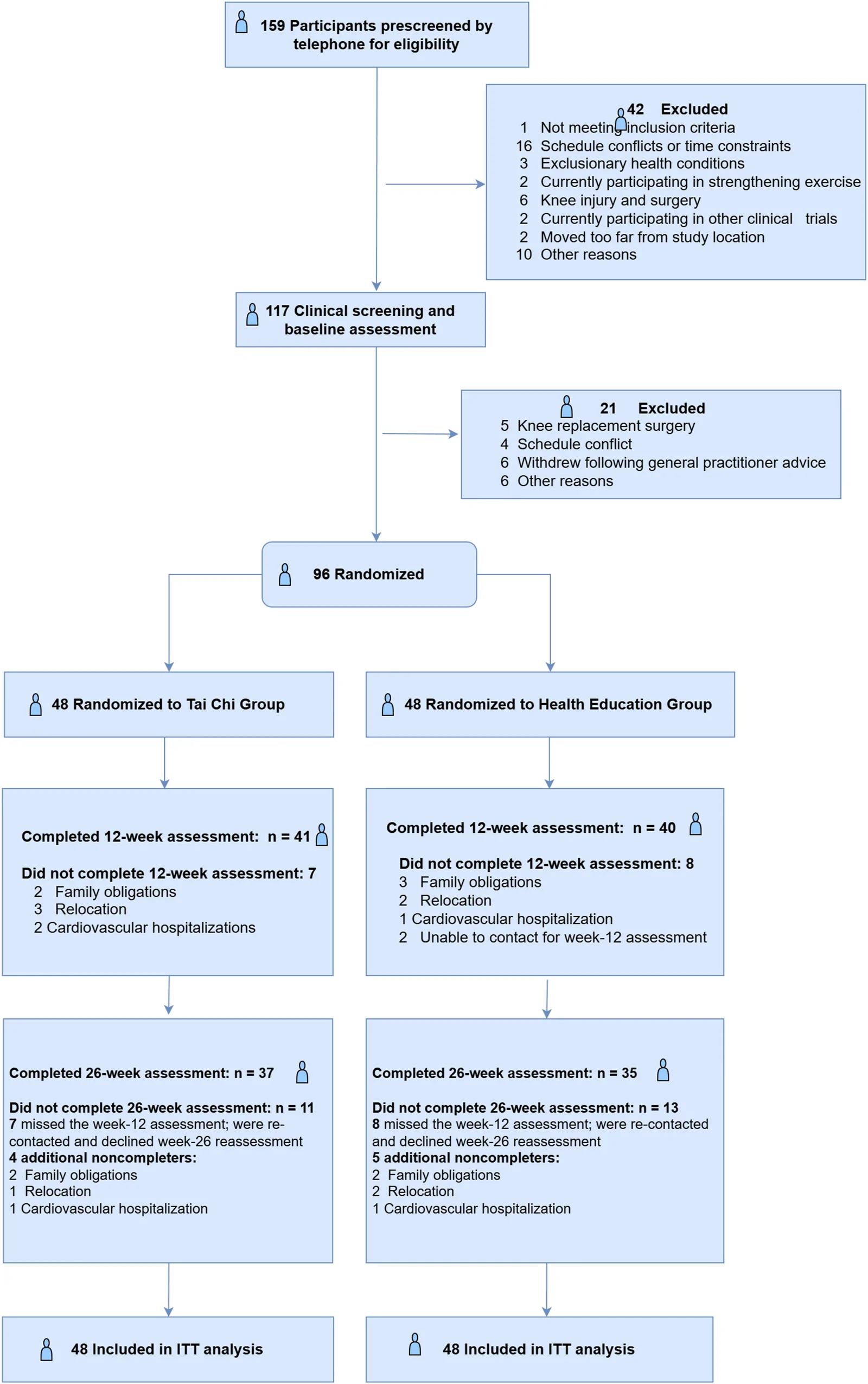
Participant flow diagram. TCG indicates TCG; HEG, health education group; ITT, intention-to-treat. The 15 participants missing the week-12 assessment were re-contacted before week 26 and declined an in-person week-26 assessment.

Secondary outcomes were knee pain during the previous week measured with a 100-mm visual analog scale (KVAS; higher scores indicate greater pain) [17], Western Ontario and McMaster Universities Osteoarthritis Index (WOMAC) total and subscale scores (pain, stiffness, and physical function; higher scores indicate worse symptoms) [18], handgrip strength, the 14-item Modified Falls Efficacy Scale (MFES; range, 0–140; higher scores indicate greater confidence), and ASMI by bioelectrical impedance analysis. Prespecified responder thresholds were reductions of at least 15 mm for KVAS [19] and at least 40 points for WOMAC total score [20].

### 2.7 Adverse events

Nonserious adverse events were actively solicited through week 12 and serious adverse events monitored through week 26. A masked study physician adjudicated seriousness and intervention relatedness from available records. Symptom-relief treatments were recorded separately.

### 2.8 Sample size calculation

The sample size was determined a priori based on the primary outcome, change in TUG time from baseline to week 12. Effect size estimates were derived from a previous study of older adults with knee osteoarthritis, in which the postintervention mean (SD) TUG times were 7.8 (0.9) seconds in the TCG and 8.6 (1.2) seconds in the control group, corresponding to a standardized mean difference (Cohen d) of 0.754 [21]. Assuming a 2-sided α of 0.05 and 90% power, 38 participants per group were required. For this calculation, the correlation between baseline and postbaseline TUG measurements was conservatively assumed to be zero; a positive correlation would be expected to reduce the variance of change scores and provide greater than 90% power. Allowing for 20% attrition, the target sample size was increased to 48 participants per group (96 participants in total).

### 2.9 Statistical analysis

The SAP (version 1.2, December 20, 2024) was finalized before recruitment. Analyses followed the intention-to-treat principle. The primary estimand was the between-group difference in TUG change from baseline to week 12. Incomplete adherence, symptom-relief treatment, and medical events or hospitalizations were handled with a treatment-policy strategy: participants remained in their randomized group and all available outcomes were analyzed.

Continuous outcomes were analyzed with restricted-maximum-likelihood linear mixed-effects models including group, categorical visit, group-by-visit interaction, site, age, sex, BMI, KL grade, and a participant-specific random intercept. Age, sex, BMI, and KL grade were prespecified a priori because of their prognostic relevance to mobility and knee osteoarthritis outcomes; site was included because randomization was stratified by site. Contrasts of model-estimated marginal means provided between-group differences in change with 95% CIs and 2-sided p-values. Week 12 was the primary endpoint; week 26 was postintervention follow-up.

Responder thresholds were improvement ≥2.8 s in TUG, reduction ≥15 mm in KVAS, and reduction ≥40 points in WOMAC total score. Modified Poisson regression with robust standard errors estimated adjusted risk ratio (RR)s and 95% CIs, adjusting for site, age, sex, BMI, and KL grade. Missing outcomes at each visit were classified as nonresponse, retaining all randomized participants in the denominator.

Primary mixed-effects models used all available observations under a missing-at-random assumption. Sensitivity analyses included complete cases, multilevel multiple imputation with 20 imputations, and delta-adjusted pattern-mixture models under missing-not-at-random (MNAR) assumptions. Delta shifts of 0.5, 1.0, and 1.5 s were applied to missing TUG values, including differential penalties applied only to the TCG.

Additional sensitivity analyses added an uncorrelated participant-specific random slope for numeric time while retaining categorical visit effects, excluded observations with absolute standardized conditional residuals >3, and used a per-protocol population. Per protocol required ≥80% supervised-session adherence, ≥80% home-practice adherence, and a week-26 assessment.

Prespecified exploratory subgroup analyses of week-12 TUG were conducted by sex, age (<75 vs ≥ 75 years), body mass index (<24 vs ≥ 24 kg/m^2^), and KL grade (I–II vs III–IV) using group-by-visit-by-subgroup interactions; interaction p-values assessed effect modification.

No multiplicity adjustment was applied to secondary, responder, sensitivity, or subgroup analyses, which were interpreted as exploratory. Tests were 2-sided. Analyses used R version 4.6.0. Additional statistical details are provided in Supplement 1, eMethods.

## 3 Results

### 3.1 Participant flow and baseline characteristics

From February to July 2025, 159 individuals were prescreened, 117 underwent clinical screening and baseline assessment, and 96 were randomized 1:1 to TCG or HEG (n = 48 each). Each site enrolled 48 participants (24 per group). All randomized participants were included in the intention-to-treat analysis (Fig. 1).

Assessments were complete through week 8. At week 12, 41 TC G participants (85.4%) and 40 HEG participants (83.3%) were assessed. All 15 week-12 noncompleters were re-contacted before week 26 and declined reassessment. A further 4 TC G and 5 HEG participants missed week 26, leaving 37 (77.1%) and 35 (72.9%) with week-26 data; cumulative week-26 noncompletion was 11 and 13, respectively (Fig. 1; Supplement 1, eTables 1 and 7).

Baseline characteristics were generally similar (Table 1). KL grade III was more common in the TCG than HEG (41.7% vs 27.1%), whereas grade II was more common in the HEG (54.2% vs 37.5%); KL grade was included in adjusted models. Baseline TUG and KVAS were similar by subsequent assessment completeness. Participants with incomplete follow-up had lower baseline WOMAC total scores than completers in both the week-12 (143.5 vs 150.3) and week-26 (144.0 vs 150.9) completeness comparisons (Supplement 1, eTable 8).

**Table 1:** Baseline characteristics of participants by randomization group.

Characteristic	TCG (n = 48)	HEG (n = 48)
Age, y	72.1 (3.2)	72.4 (3.1)
Sex, n (%)		
Female	16 (33.3%)	18 (37.5%)
Male	32 (66.7%)	30 (62.5%)
Study site, n (%)		
XS	24 (50.0%)	24 (50.0%)
YY	24 (50.0%)	24 (50.0%)
BMI, kg/m^2^	22.0 (2.7)	22.5 (3.6)
Kellgren-Lawrence grade, n (%)		
Grade I	6 (12.5%)	5 (10.4%)
Grade II	18 (37.5%)	26 (54.2%)
Grade III	20 (41.7%)	13 (27.1%)
Grade IV	4 (8.3%)	4 (8.3%)
Timed Up and Go test, s	13.1 (2.1)	13.2 (2.3)
Knee Visual Analog Scale, mm	59.3 (6.7)	60.9 (6.6)
WOMAC total score, points	148.6 (10.5)	149.8 (8.8)
WOMAC pain, points	37.8 (3.2)	37.6 (4.1)
WOMAC stiffness, points	15.0 (2.6)	15.2 (2.2)
WOMAC physical function, points	95.8 (10.3)	97.0 (8.8)
Grip strength, kg	23.9 (2.0)	23.7 (2.2)
Modified Falls Efficacy Scale, points	102.1 (11.0)	101.8 (10.9)
Appendicular skeletal muscle mass index, kg/m^2^	6.2 (1.5)	6.2 (1.4)

Note: Data are mean (SD) for continuous variables and n (%) for categorical variables. No baseline hypothesis tests were performed.

Abbreviations: BMI, body mass index; HEG, health education group; TCG, Tai Chi group; WOMAC, Western Ontario and McMaster Universities Osteoarthritis Index; XS, Xiangshan Hospital of Traditional Chinese Medicine; YY, Yuyuan Community Health Service Center.

### 3.2 Primary outcome

At week 12, adjusted mean TUG change was −3.36 s (95% CI, −4.00 to −2.72) in the TCG and −0.84 s (95% CI, −1.48 to −0.19) in the HEG. The adjusted between-group difference was −2.52 s (95% CI, −3.43 to −1.61; p < 0.001), favoring TCG (Table 2; Fig. 2), but slightly below the prespecified 2.8-s individual improvement threshold.

**Table 2:** Adjusted changes from baseline and between-group differences in continuous outcomes at weeks 12 and 26.

Outcome	Visit	TCG, adjusted change from baseline (95% CI)	HEG, adjusted change from baseline (95% CI)	Adjusted between-group(95% CI)	p-value
Timed Up and Go test, s	Week 12	−3.36 (−4.00 to −2.72)	−0.84 (−1.48 to −0.19)	−2.52 (−3.43 to −1.61)	<0.001
Timed Up and Go test, s	Week 26	−3.01 (−3.67 to −2.35)	−1.55 (−2.22 to −0.87)	−1.46 (−2.41 to −0.52)	0.002
Knee Visual Analog Scale, mm	Week 12	−23.28 (−25.73 to −20.84)	−20.47 (−22.93 to −18.01)	−2.82 (−6.28 to 0.65)	0.111
Knee Visual Analog Scale, mm	Week 26	−19.06 (−21.57 to −16.54)	−18.25 (−20.81 to −15.69)	−0.81 (−4.39 to 2.78)	0.659
WOMAC total score, points	Week 12	−54.03 (−58.86 to −49.20)	−47.23 (−52.09 to −42.37)	−6.80 (−13.65 to 0.05)	0.052
WOMAC total score, points	Week 26	−43.69 (−48.66 to −38.72)	−34.39 (−39.44 to −29.34)	−9.30 (−16.38 to −2.21)	0.010
WOMAC pain, points	Week 12	−21.09 (−22.54 to −19.63)	−17.86 (−19.33 to −16.40)	−3.22 (−5.29 to −1.16)	0.002
WOMAC pain, points	Week 26	−23.61 (−25.11 to −22.11)	−19.90 (−21.43 to −18.38)	−3.71 (−5.84 to −1.57)	<0.001
WOMAC stiffness, points	Week 12	−8.22 (−8.90 to −7.54)	−8.13 (−8.81 to −7.44)	−0.09 (−1.06 to 0.88)	0.855
WOMAC stiffness, points	Week 26	−8.04 (−8.74 to −7.33)	−7.36 (−8.07 to −6.65)	−0.68 (−1.68 to 0.32)	0.184
WOMAC physical function, points	Week 12	−24.73 (−29.35 to −20.11)	−21.24 (−25.89 to −16.59)	−3.49 (−10.04 to 3.07)	0.296
WOMAC physical function, points	Week 26	−12.04 (−16.79 to −7.29)	−7.13 (−11.96 to −2.29)	−4.91 (−11.69 to 1.86)	0.155
Grip strength, kg	Week 12	1.90 (1.46–2.34)	1.73 (1.28–2.18)	0.17 (−0.46 to 0.80)	0.599
Grip strength, kg	Week 26	2.89 (2.43–3.35)	2.72 (2.25–3.19)	0.17 (−0.48 to 0.83)	0.606
Modified Falls Efficacy Scale, points	Week 12	26.19 (25.09–27.28)	24.47 (23.37–25.57)	1.72 (0.17–3.27)	0.030
Modified Falls Efficacy Scale, points	Week 26	23.72 (22.59–24.85)	21.37 (20.22–22.52)	2.35 (0.73–3.96)	0.004
Appendicular skeletal muscle mass index, kg/m^2^	Week 12	0.418 (0.357–0.479)	0.508 (0.446–0.569)	−0.089 (−0.176 to −0.003)	0.043

Note: Models included randomization group, categorical visit, group-by-visit interaction, study site, age, sex, body mass index, and Kellgren-Lawrence grade, with a participant-specific random intercept.

Changes and between-group differences were estimated using model-based contrasts from the full estimated covariance matrix. Between-group differences are TCG minus HEG differences in change from baseline.

Negative values favor TCG for TUG, KVAS, and WOMAC outcomes; positive values favor TCG for grip strength, MFES, and ASMI.

ASMI model estimates are displayed to 3 decimal places because a 2-decimal display would round a confidence-limit value close to zero to 0.00.

The primary hypothesis concerns TUG at week 12. No multiplicity adjustment was applied to secondary outcomes or other time points; these analyses are exploratory.

Abbreviations: ASMI, appendicular skeletal muscle mass index; CI, confidence interval; HEG, health education group; KVAS, knee visual analog scale; MFES, Modified Falls Efficacy Scale; TCG, Tai Chi group; TUG, Timed Up and Go; WOMAC, Western Ontario and McMaster Universities Osteoarthritis Index.

**Fig. 2:**
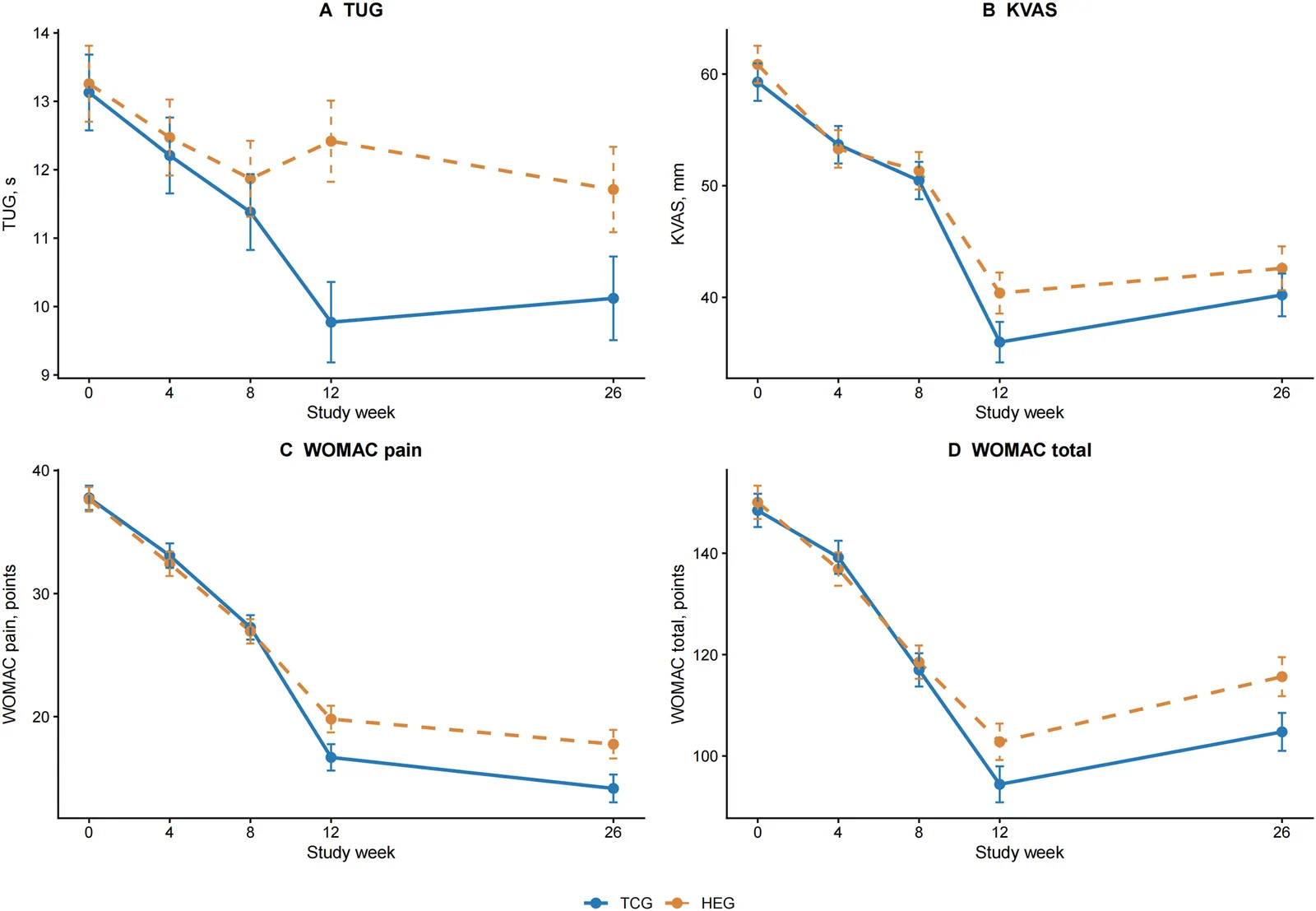
Model-estimated trajectories for (A) TUG, (B) KVAS, (C) WOMAC pain, and (D) WOMAC total. Points and 95% CIs are estimated marginal means from the same site-adjusted categorical-time mixed models used for Table 2.

At week 26, adjusted mean TUG change was −3.01 s in the TCG and −1.55 s in the HEG, for a between-group difference of −1.46 s (95% CI, −2.41 to −0.52; p = 0.002). Fig. 2 shows model-estimated trajectories. Adjusted results across all follow-up visits are provided in Supplement 1, eTable 2.

### 3.3 minimal clinically important difference responder analyses

At week 12, 28 of 48 participants (58.3%) in the TCG and 6 of 48 (12.5%) in the HEG achieved an improvement of at least 2.8 s in TUG time. The covariate-adjusted RR for achieving this responder threshold was 4.81 (95% CI, 2.29–10.07; p < 0.001), favoring the TCG. At week 26, 17 of 48 participants (35.4%) in the TCG and 8 of 48 (16.7%) in the HEG met the TUG responder criterion, corresponding to an adjusted RR of 2.12 (95% CI, 1.07–4.20; p = 0.031) (Fig. 3; Supplement 1, eTable 3).

**Fig. 3:**
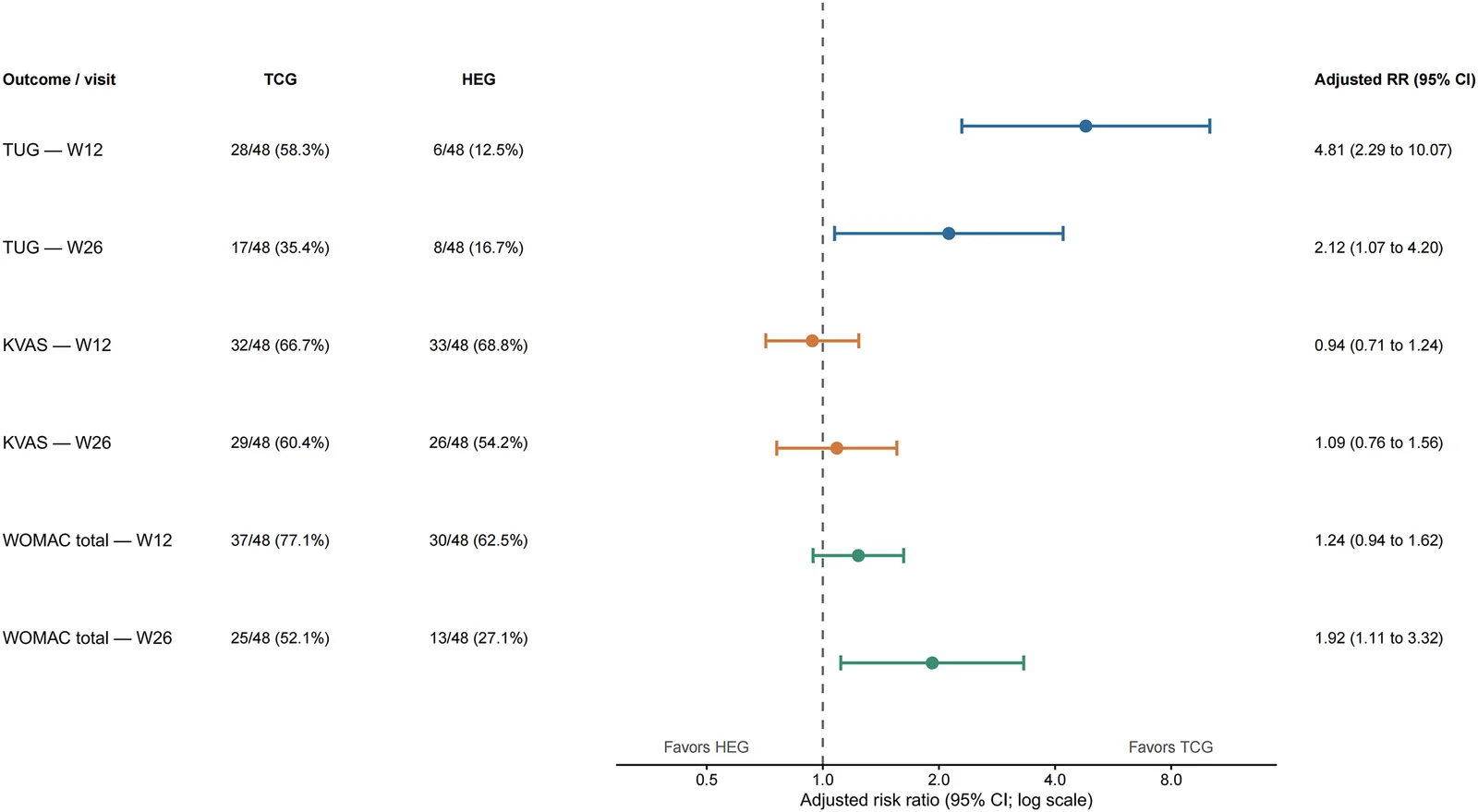
Adjusted RRs for achieving prespecified minimal clinically important difference thresholds at weeks 12 and 26. Thresholds were TUG reduction ≥2.8 s, KVAS reduction ≥15 mm, and WOMAC total reduction ≥40 points. Missing follow-up assessments were classified as nonresponse. RRs were adjusted for study site, age, sex, BMI, and KL grade; RR > 1 favors TCG. Abbreviations: BMI, body mass index; KL, Kellgren-Lawrence grade; KVAS, knee visual analog scale; MCID, minimal clinically important difference; RR, risk ratio; TCG, Tai Chi group; TUG, Timed Up and Go; WOMAC, Western Ontario and McMaster Universities Osteoarthritis Index.

**Table 3:** Adverse events and symptom-relief treatments.

Measure	TCG, n/48 (%)	HEG, n/48 (%)
Participants reporting ≥1 nonserious AE, weeks 0–12	6/48 (12.5)	3/48 (6.3)
Transient knee pain exacerbation	4/48 (8.3)	0/48 (0)
Ankle sprain	2/48 (4.2)	0/48 (0)
Thigh muscle soreness	0/48 (0)	3/48 (6.3)
Participants with ≥1 SAE through week 26	3/48 (6.3)	2/48 (4.2)
Participants using ≥1 symptom-relief treatment, weeks 0–12	6/48 (12.5)	3/48 (6.3)
Etoricoxib	6/48 (12.5)	3/48 (6.3)
Topical herbal heat compress	0/48 (0)	3/48 (6.3)

Note: Nonserious AEs were collected during weeks 0–12. SAEs were monitored through week 26. All SAEs were cardiovascular hospitalizations and were judged unrelated or unlikely related to the interventions based on available clinical records.

Symptom-relief treatments were recorded separately from adverse events. Treatment categories were not mutually exclusive. Abbreviations: AE, adverse event; HEG, health education group; SAE, serious adverse event; TCG, Tai Chi group.

KVAS responder proportions were similar at week 12 (66.7% vs 68.8%; adjusted RR, 0.94; 95% CI, 0.71–1.24) and week 26 (60.4% vs 54.2%; adjusted RR, 1.09; 95% CI, 0.76–1.56). WOMAC total response was 77.1% vs 62.5% at week 12 (adjusted RR, 1.24; 95% CI, 0.94–1.62) and 52.1% vs 27.1% at week 26 (adjusted RR, 1.92; 95% CI, 1.11–3.32). Missing outcomes were classified as nonresponse.

### 3.4 Secondary outcomes

WOMAC pain favored TCG at week 12 (adjusted difference, −3.22 points; 95% CI, −5.29 to −1.16) and week 26 (−3.71; 95% CI, −5.84 to −1.57) (Table 2; Fig. 2). WOMAC total differences were −6.80 points (95% CI, −13.65 to 0.05) at week 12 and −9.30 (95% CI, −16.38 to −2.21) at week 26. KVAS differences were small at week 12 (−2.82 mm; 95% CI, −6.28 to 0.65) and week 26 (−0.81 mm; 95% CI, −4.39 to 2.78). WOMAC stiffness, physical function, and grip strength showed no clear between-group differences.

MFES improved modestly more in TCG at week 12 (adjusted difference, 1.72 points; 95% CI, 0.17 to 3.27) and week 26 (2.35; 95% CI, 0.73 to 3.96). ASMI increased in both groups at week 12, slightly more in HEG (adjusted difference, −0.089 kg/m^2^; 95% CI, −0.176 to −0.003). Secondary outcomes were exploratory because no multiplicity adjustment was applied. Additional model-estimated secondary-outcome trajectories are shown in Supplement 1, eFig. 2.

### 3.5 Sensitivity and subgroup analyses

Primary TUG estimates were similar in complete-case and multiple-imputation analyses (Supplement 1, eTable 9). Under the most unfavorable differential MNAR scenario, adding 1.5 s only to missing TCG values, the between-group difference remained −2.32 s (95% CI, −3.22 to −1.41) at week 12 and −1.12 s (95% CI, −2.07 to −0.17) at week 26. The delta-adjusted MNAR scenarios are also displayed in Supplement 1, eFig. 4.

Results were materially unchanged with the random-slope sensitivity model and after excluding observations with absolute standardized conditional residuals >3. In the per-protocol population, the between-group difference favored TCG at week 12 (−2.27 s; 95% CI, −3.69 to −0.86) but was smaller and imprecise at week 26 (−0.68 s; 95% CI, −2.09 to 0.73). These analyses are summarized in Supplement 1, eTable 10.

Prespecified exploratory subgroup analyses showed no clear heterogeneity in week-12 TUG effects by sex, age, BMI, or KL grade (all interaction p > 0.29; Supplement 1, eTable 11 and eFig. 3).

### 3.6 Safety and symptom-relief treatments

During weeks 0–12, 6 TC G and 3 HEG participants experienced ≥1 nonserious AE (Supplement 1, eTable 6): 4 transient knee pain exacerbations and 2 ankle sprains in TCG, and 3 cases of thigh soreness in HEG. No intervention-related AE caused withdrawal.

Five serious adverse events occurred through week 26 (TCG, 3; HEG, 2), all cardiovascular hospitalizations: 3 during weeks 0–12 and 2 during weeks 12–26. None was judged intervention-related.

During weeks 0–12, symptom-relief treatment was used by 6 TC G and 3 HEG participants. All used etoricoxib; the same 3 HEG participants also used topical herbal heat compresses. Treatment categories overlapped (Supplement 1, eTable 6).

### 3.7 Intervention adherence and fidelity

Adherence to supervised sessions and home practice was generally high, although the proportions meeting the prespecified 80% threshold differed between intervention components (Supplement 1, eTable 4). Participants in the TCG attended a mean (SD) of 3.81 (0.39) of 4 prescribed supervised sessions, corresponding to a mean adherence of 95.3% (9.9%); 39 of 48 participants (81.3%) attended at least 80% of supervised sessions. Participants in the HEG attended a mean (SD) of 5.67 (0.52) of 6 prescribed sessions, corresponding to 94.4% (8.7%) adherence; 47 of 48 participants (97.9%) met the 80% supervised-session threshold.

For 50 prescribed home-practice days, mean (SD) completed days were 43.10 (4.26) in TCG and 39.71 (4.59) in HEG, corresponding to 86.2% and 79.4% adherence; 36/48 (75.0%) and 23/48 (47.9%), respectively, achieved ≥80%. Mean session duration was 29.4 (5.8) and 24.7 (6.1) minutes, and 42 (87.5%) and 45 (93.8%) completed the planned final phase (Supplement 1, eTable 5).

## 4 Discussion

In this 2-site randomized clinical trial of older adults with sarcopenic knee osteoarthritis, a 12-week simplified 8-form Tai Chi program produced greater improvement in mobility than health education plus progressive home exercise. The adjusted between-group difference in TUG change was −2.52 s at week 12 and −1.46 s at week 26. Although the mean week-12 difference was slightly smaller than the prespecified 2.8-s threshold used to define clinically important improvement at the individual level, participants assigned to Tai Chi were substantially more likely to achieve this threshold at both week 12 and week 26.

The improvement in TUG is broadly consistent with previous systematic reviews and meta-analyses suggesting that Tai Chi can improve physical function, balance, and walking-related outcomes in people with knee osteoarthritis and in older adults [[22], [23], [24]]. Randomized trials have also shown functional benefits of Tai Chi in knee osteoarthritis, including comparisons with hydrotherapy and conventional physical therapy [25,26]. An important feature of the present trial is the comparator. Participants in the HEG did not receive usual care or attention control alone; they received repeated health education and, from weeks 3–12, a progressive home program incorporating lower-extremity strengthening, functional mobility, balance, stair practice, and cognitive-motor tasks. This active comparator is clinically relevant because structured exercise and strengthening interventions can themselves improve function in knee osteoarthritis [27]. Therefore, the observed between-group differences are more appropriately interpreted as the added benefit of the Tai Chi program relative to an active self-management and exercise intervention rather than as an effect relative to no exercise. This may also partly explain why both groups improved over time on several outcomes.

The pattern of secondary outcomes was not uniform. WOMAC pain favored Tai Chi at weeks 12 and 26, whereas the single-item KVAS showed little between-group separation. WOMAC total score also favored Tai Chi at week 26, although the week-12 estimate was less precise and its confidence interval included zero. Likewise, the clear improvement in performance-based TUG was not mirrored by clear between-group differences in the WOMAC stiffness or physical-function subscales, suggesting that performance-based mobility and self-reported symptom/function measures captured different aspects of treatment response. These differences may reflect the fact that the pain measures capture related but not identical aspects of the symptom experience: a single visual analog scale rating provides a global measure of pain intensity, whereas the WOMAC pain subscale assesses pain across several activities [17,18]. The trial was not designed to determine why the instruments produced different patterns, and the absence of multiplicity adjustment means that secondary outcomes should be interpreted as exploratory. Similarly, the modest differences in MFES favored Tai Chi but lacked a prespecified threshold for clinical importance.

The mobility benefit was not accompanied by a greater increase in ASMI. ASMI increased in both groups at week 12, with a slightly greater increase in the HEG. This finding argues against interpreting the TUG improvement as evidence that Tai Chi increased muscle mass. The active control program included strengthening exercises and dietary education, which could plausibly have contributed to changes in body composition. More broadly, Tai Chi may improve mobility through enhanced postural control, proprioception, and sensorimotor coordination rather than through gains in muscle mass alone [23,28,29]. Pain-related adaptations in movement strategy may provide an additional pathway through which mobility changes occur [30]. These potential mechanisms were not directly measured and should therefore remain hypotheses for future study rather than causal explanations of the present findings.

The responder analyses provide a complementary perspective on the clinical interpretation of the primary outcome. The 2.8-s TUG threshold was prespecified from previous work in knee osteoarthritis and was applied to within-participant improvement rather than as a required threshold for the between-group mean difference [16]. Accordingly, the finding that the adjusted mean difference at week 12 was −2.52 s does not conflict with the observation that 58.3% of participants in the TCG, compared with 12.5% in the HEG, achieved an improvement of at least 2.8 s. The adjusted RR for response was 4.81 at week 12 and remained above 1 at week 26. Mean treatment effects and responder analyses answer different clinical questions and are therefore best interpreted together rather than using one to substitute for the other.

Several aspects of the analysis support the robustness of the primary result. Randomization was stratified by site, outcome assessment was masked, and the SAP was finalized before recruitment. The primary model treated visit as categorical and adjusted for site and prespecified prognostic covariates. Sensitivity analyses using complete cases, multilevel multiple imputation, pattern-mixture models under MNAR assumptions, an alternative random-slope specification, and exclusion of outlying observations produced results that were materially similar to the primary analysis. Even under the most unfavorable differential MNAR scenario examined, in which a 1.5-s penalty was applied only to missing TUG values in the TCG, the estimated between-group differences remained in favor of Tai Chi at weeks 12 and 26. Nevertheless, 15 participants lacked week-12 assessments and 24 lacked week-26 assessments, and sensitivity analyses cannot establish that the missing-at-random assumption was correct. The per-protocol analysis supported the week-12 finding but was less precise and did not show a clear difference at week 26. Prespecified subgroup analyses also showed no clear evidence that the week-12 TUG effect differed according to sex, age, BMI, or KL grade.

This study has several limitations. First, the sample size was modest and the trial was powered for the week-12 TUG outcome rather than for secondary outcomes, subgroup interactions, or safety events. Second, participants were recruited from 2 sites within a single urban district in Shanghai, which may limit generalizability to other settings and populations. Third, follow-up was limited to 26 weeks and outcome completeness declined over time. Fourth, home-practice adherence relied partly on participant-reported logs, and the 2 intervention groups differed in both content and contact schedules, preventing attribution of the observed effect to any single component of Tai Chi. Fifth, no multiplicity adjustment was applied to secondary, responder, sensitivity, or subgroup analyses, and these findings should therefore be interpreted as exploratory. Finally, although trial registration was submitted before enrollment began, the registry record became publicly available only after recruitment had started because of administrative processing.

Overall, simplified 8-form Tai Chi improved mobility compared with an active health education and home-exercise program in older adults with sarcopenic knee osteoarthritis. The average week-12 TUG advantage was slightly smaller than the prespecified threshold for clinically important individual improvement, but participants receiving Tai Chi were more likely to achieve that threshold, and a smaller average benefit remained at week 26. The findings support Tai Chi as a potentially useful mobility-focused intervention in this population, while larger multicenter trials with longer follow-up are needed to confirm durability, clarify mechanisms, and better define the magnitude of clinically important benefit.

## CRediT author statement

**Guang-Ming Liu:** Conceptualization; Methodology; Investigation; Writing – original draft; Writing – review & editing.

**Li-Ping Chai:** Conceptualization; Methodology; Investigation; Writing – original draft; Writing – review & editing.

**Ping Huang:** Conceptualization; Methodology; Investigation; Writing – original draft; Writing – review & editing; Supervision.

**Jia-Yu Yang:** Investigation; Formal analysis; Writing – original draft.

**Wei-Yi Zhan:** Investigation; Resources.

**Chen-Yi Rong:** Investigation; Resources.

**Lin-Fei Yang:** Investigation; Resources; Writing – review & editing.

**Jiao-Jie Zha:** Investigation; Formal analysis; Writing – original draft.

**Wen-Ting Zhang:** Investigation; Resources; Writing – review & editing.

**Bo Sun:** Conceptualization; Methodology; Funding acquisition; Supervision; Writing – review & editing.

**Hong-Sheng Zhan:** Conceptualization; Methodology; Resources; Funding acquisition; Supervision; Writing – review & editing.

**Equal contribution:** Guang-Ming Liu, Li-Ping Chai, and Ping Huang contributed equally as co-first authors.

**Correspondence:** Hong-Sheng Zhan and Bo Sun served as co-corresponding authors.

## Data availability

Deidentified participant data and statistical code will be made available upon reasonable request to the corresponding authors, beginning 6 months after publication and ending 5 years after publication.

## Disclaimer

The views expressed in this article are those of the authors and do not necessarily reflect the official positions of the funding agencies.

## Additional contributions

We gratefully acknowledge the clinical and administrative staff at the participating institutions for their support in participant recruitment and intervention delivery. We also thank the research coordinators and assessors who contributed to data collection and quality control.

## Declaration of Generative AI and AI-assisted technologies in the writing proces

During the preparation of this work, the authors used ChatGPT (OpenAI) to assist with language editing and manuscript clarity. After using this tool, the authors reviewed and edited the content as needed and take full responsibility for the content of the article.

## Funding/support

• National Natural Science Foundation of China (Grant No. 82074466);
• National Natural Science Foundation of China (Grant No. 82374481);
• Construction Project of Zhan Hongsheng's National Famous and Veteran Traditional Chinese Medicine Experts Inheritance Studio (Document No. [2022] No. 75 issued by the Department of Personnel Education of the National Administration of Traditional Chinese Medicine);
• Zhan Hongsheng Traditional Chinese Medicine Inheritance and Innovation Studio (Grant No. 2025CXGZS-03);
• 2023–2026 Talent Echelon Construction Project for Professional Personnel in Huangpu District Health System (Grant No. 2023BJ05);
• Clinical and Biomechanical Mechanism Study on Eight-Style Da She Daoyin in the Treatment of Sarcopenic Osteoarthritis in the Elderly, Huangpu District Scientific Research Project (Grant No. HLM202420);
• Sun Bo Famous Doctor Studio in Huangpu District (Grant No. 2023MY05);
• Key Discipline Project of Traditional Chinese Medicine Orthopedics and Traumatology in Huangpu District Health System (Led by Sun Bo) (Grant No. 2025ZDXK05); and
• Liu Guangming Staff Innovation Studio in Huangpu District.

The funding organizations had no role in the design and conduct of the study; collection, management, analysis, and interpretation of the data; preparation, review, or approval of the manuscript; or decision to submit the manuscript for publication.

## Conflict of interest

None reported.

## Abbreviations

AE: adverse event
ASMI: appendicular skeletal muscle mass index
BMI: body mass index
CI: confidence interval
CONSORT: Consolidated Standards of Reporting Trials
HEG: health education group
ITT: intention-to-treat
ITMCTR: International Traditional Medicine Clinical Trial Registry
KL: Kellgren-Lawrence
KVAS: knee visual analog scale
MCID: minimal clinically important difference
MFES: Modified Falls Efficacy Scale
MNAR: missing not at random
RR: risk ratio
SAE: serious adverse event
SAP: statistical analysis plan
SD: standard deviation
SPKOA: sarcopenic knee osteoarthritis
TCG: Tai Chi group
TUG: Timed Up and Go
WOMAC: Western Ontario and McMaster Universities Osteoarthritis Index

## References

[1] Cruz-Jentoft A.J., Sayer A.A. (2019). Sarcopenia. Lancet.

[2] Hunter D.J., Bierma-Zeinstra S. (2019). Osteoarthritis. Lancet.

[3] Chen T.X., Yang T.J., Cheng S., Zhang Z.L., Liu T. (2025). High prevalence and multifactorial risks of sarcopenia in knee osteoarthritis: a systematic review and meta-analysis. J. Orthop. Surg..

[4] Liu G.M., Sun B., Yang J.Y., Rong C.Y., Wang Y., Hu Y.M. (2025). Development and validation of the BPDKC model for predicting sarcopenic knee osteoarthritis in community-dwelling older adults: a cross-sectional study. BMC Muscoskelet. Disord..

[5] Bannuru R.R., Osani M.C., Vaysbrot E.E., Arden N.K., Bennell K., Bierma-Zeinstra S.M.A. (2019). OARSI guidelines for the non-surgical management of knee, hip, and polyarticular osteoarthritis. Osteoarthr. Cartil..

[6] Shen Y., Shi Q., Nong K., Li S., Yue J., Huang J. (2023). Exercise for sarcopenia in older people: a systematic review and network meta-analysis. J. Cachexia Sarcopenia Muscle.

[7] Wang C., Schmid C.H., Hibberd P.I., Kalish R., Roubenoff R., Rones R. (2009). Tai chi is effective in treating knee osteoarthritis: a randomized controlled trial. Arthritis Rheum..

[8] Wehner C., Blank C., Arvandi M., Wehner C., Schobersberger W. (2021). Effect of Tai Chi on muscle strength, physical endurance, postural balance and flexibility: a systematic review and meta-analysis. BMJ Open Sport Exerc Med.

[9] Zhu G.C., Chen K.M., Belcastro F. (2024). Effects of different mind-body exercises in managing chronic pain of older adults: a network meta-analysis of randomized trials. Geriatr Nurs N Y N.

[10] Qiao H., Hao X., Wang G. (2024). Effects of mind-body exercise on knee osteoarthritis: a systematic review and meta-analysis of randomized controlled trials. BMC Muscoskelet. Disord..

[11] Liao C.D., Huang S.W., Chen H.C., Huang Y.Y., Liou T.H., Lin C.L. (2023). Effects of protein supplementation combined with resistance exercise training on walking speed recovery in older adults with knee osteoarthritis and sarcopenia. Nutrients.

[12] Hopewell S., Chan A.W., Collins G.S., Hróbjartsson A., Moher D., Schulz K.F. (2025). CONSORT 2025 statement: updated guideline for reporting randomised trials. BMJ.

[13] Chen L.K., Woo J., Assantachai P., Auyeung T.W., Chou M.Y., Iijima K. (2020). Asian working group for sarcopenia: 2019 consensus update on sarcopenia diagnosis and treatment. J. Am. Med. Dir. Assoc..

[14] Kellgren J.H., Lawrence J.S. (1957). Radiological assessment of osteo-arthrosis. Ann. Rheum. Dis..

[15] Podsiadlo D., Richardson S. (1991). The timed “up & go”: a test of basic functional mobility for frail elderly persons. J. Am. Geriatr. Soc..

[16] Mostafaee N., Pirayeh N., Fakoor M. (2024). Responsiveness and minimal clinically important changes of common patient-reported and performance-based outcome measures of physical function in patients with knee osteoarthritis. Physiother. Theory Pract..

[17] Hawker G.A., Mian S., Kendzerska T., French M. (2011). Measures of adult pain: visual analog scale for pain (VAS pain), numeric rating scale for pain (NRS pain), McGill pain questionnaire (MPQ), short-form McGill pain questionnaire (SF-MPQ), chronic pain grade scale (CPGS), short form-36 bodily pain scale (SF-36 BPS), and measure of intermittent and constant osteoarthritis pain (ICOAP). Arthritis Care Res..

[18] Bellamy N., Buchanan W., Goldsmith C., Campbell J., Stitt L. (1988). Validation study of WOMAC: a health status instrument for measuring clinically important patient relevant outcomes to antirheumatic drug therapy in patients with osteoarthritis of the hip or knee. J. Rheumatol..

[19] Bellamy N., Carette S., Ford P.M., Kean W.F., Le Riche N.G., Lussier A. (1992). Osteoarthritis antirheumatic drug trials. III. Setting the Delta for clinical trials--results of a consensus development (delphi) exercise. J. Rheumatol..

[20] Kim M.S., Koh I.J., Choi K.Y., Sung Y.G., Park D.C., Lee H.J. (2021). The minimal clinically important difference (MCID) for the WOMAC and factors related to achievement of the MCID after medial opening wedge high tibial osteotomy for knee osteoarthritis. Am. J. Sports Med..

[21] Li J., Cheng L. (2019). The effect of Taichi and resistance training on osteoarthritis symptoms of the elderly and the exercise capacity. Chin. J. Rehabil. Med..

[22] Kelley Ga, Kelley Ks, Callahan Lf (2022). Clinical relevance of Tai Chi on pain and physical function in adults with knee osteoarthritis: an ancillary meta-analysis of randomized controlled trials. Sci. Prog..

[23] You Y., Liu J., Tang M., Wang D., Ma X. (2021). Effects of Tai Chi exercise on improving walking function and posture control in elderly patients with knee osteoarthritis: a systematic review and meta-analysis. Med Baltim.

[24] Bertolini R., Vercelino R., Ferreira L.F., Telles da Rosa L.H. (2025). Effects of Tai Chi chuan on older adults' balance: a systematic review with meta-analysis. J. Aging Phys. Activ.

[25] Wang C., Schmid C.H., Iversen M.D., Harvey W.F., Fielding R.A., Driban J.B. (2016). Comparative effectiveness of Tai Chi versus physical therapy for knee osteoarthritis: a randomized trial. Ann. Intern. Med..

[26] Fransen M., Nairn L., Winstanley J., Lam P., Edmonds J. (2007). Physical activity for osteoarthritis management: a randomized controlled clinical trial evaluating hydrotherapy or Tai Chi classes. Arthritis Rheum..

[27] Messier S.P., Mihalko S.L., Beavers D.P., Nicklas B.J., DeVita P., Carr J.J. (2021). Effect of high-intensity strength training on knee pain and knee joint compressive forces among adults with knee osteoarthritis: the START randomized clinical trial. JAMA.

[28] Zhu Q., Huang L., Li J., Mao L., Zhang Y., Min F. (2017). Effect of taijiquan practice versus wellness education on knee proprioception in patients with knee osteoarthritis: a randomized controlled trial. J. Tradit. Chin. Med..

[29] Fu S., Yang F., Hou M., Ma Y., Liu B., Lin Z. (2026). Comparative efficacy of Tai Chi versus balance training on functional performance and postural control during sit and stand transitions in individuals with knee osteoarthritis: a randomized trial. J. Sci. Med. Sport.

[30] Hodges P.W., Tucker K. (2011). Moving differently in pain: a new theory to explain the adaptation to pain. Pain.

