# Supplementary material for: Effect of 8-form Tai Chi vs health education on mobility in older adults with sarcopenic knee osteoarthritis: A randomized clinical trial
Source: Osteoarthr Cartil Open. 2026 Sep 23;8(4):100881. doi: 10.1016/j.ocarto.2026.100881 (PMC13675917; doi:10.1016/j.ocarto.2026.100881)
Supplement: Supplementary file 1 [file mmc1.docx]

Supplement 1

**eMethods, eResults, eTables, eFigures, and eAppendix**

**Effect of 8-Form Tai Chi vs Health Education on Mobility in Older Adults With Sarcopenic Knee Osteoarthritis: A Randomized Clinical Trial**

Manuscript No. OCARTO-D-26-00089

# Contents

eMethods. Additional Statistical and Procedural Details

eResults. Additional Trial Results

eTables 1–11

eFigures 1–4

eAppendix. Intervention Details for the Tai Chi and Health Education Groups

# eMethods. Additional Statistical and Procedural Details

## eMethods 1. Analysis Population, Estimand, and Primary Model

Analyses followed the intention-to-treat principle. The primary estimand was the between-group difference in change in Timed Up and Go (TUG) time from baseline to week 12 among all randomized participants. Incomplete intervention adherence, symptom-relief treatment, and postrandomization medical events or hospitalizations were handled using a treatment-policy strategy: participants remained in their randomized group and all available outcome data were analyzed regardless of these events.

Continuous outcomes were analyzed using linear mixed-effects models fitted by restricted maximum likelihood. Fixed effects included treatment group, visit as a categorical variable (baseline and weeks 4, 8, 12, and 26), and the group-by-visit interaction, with adjustment for study site, age, sex, body mass index (BMI), and Kellgren–Lawrence (KL) grade. A participant-specific random intercept accounted for repeated measurements. Between-group differences in change from baseline were estimated using model-based contrasts of estimated marginal means. ASMI was assessed at baseline and week 12 only.

Week 12 was the primary endpoint. Week 26 was considered postintervention follow-up. No multiplicity adjustment was applied to secondary outcomes, responder analyses, sensitivity analyses, or subgroup analyses; these analyses were interpreted as exploratory.

## eMethods 2. Responder Analyses

Prespecified responder thresholds were an improvement of at least 2.8 seconds in TUG time, a reduction of at least 15 mm in knee visual analog scale (KVAS) pain, and a reduction of at least 40 points in WOMAC total score. Participants with a missing outcome at the relevant follow-up visit were classified as nonresponders, retaining all 48 randomized participants per group in the denominator. Adjusted risk ratios were estimated using modified Poisson regression with heteroskedasticity-robust standard errors and adjustment for study site, age, sex, BMI, and KL grade.

## eMethods 3. Missing Data and Sensitivity Analyses

The likelihood-based primary mixed-effects model assumes outcomes are missing at random (MAR) conditional on observed outcomes and model covariates. Missing-data sensitivity analyses included complete-case analysis, multilevel multiple imputation under MAR, and delta-adjusted pattern-mixture analyses under missing-not-at-random (MNAR) assumptions.

Multiple imputation used 20 imputed data sets generated with the mice 2l.pan method in long format, treating participant as the clustering variable. Treatment-by-visit terms and the analysis covariates were included in the imputation model, and estimates were combined using Rubin's rules.

For MNAR analyses, positive delta values of 0.5, 1.0, and 1.5 seconds were added to originally missing follow-up TUG values after MAR imputation. Equal-penalty scenarios applied the same delta to both randomized groups; differential scenarios applied the penalty only to the Tai Chi group, providing assumptions deliberately unfavorable to Tai Chi.

Additional sensitivity analyses retained categorical fixed visit effects while adding an uncorrelated participant-specific random slope for numeric time scaled in 4-week units; refitted the primary model after excluding observations with an absolute standardized conditional residual greater than 3; and used the per-protocol (PP) population defined in SAP version 1.2 as at least 80% supervised-session adherence, at least 80% home-practice adherence, and completion of the week-26 assessment.

## eMethods 4. Prespecified Exploratory Subgroup Analyses

Prespecified exploratory subgroup analyses evaluated the week-12 TUG treatment effect by sex, age (<75 vs ≥75 years), BMI (<24 vs ≥24 kg/m²), and KL grade (I–II vs III–IV). For each subgroup factor, the model included treatment group, categorical visit, subgroup, all corresponding 2-way and 3-way interaction terms, study site, the remaining prespecified baseline covariates, and a participant-specific random intercept. Interaction p values assessed evidence of treatment-effect heterogeneity.

## eMethods 5. Adherence, Fidelity, and Safety

Supervised-session adherence was calculated as sessions attended divided by sessions prescribed (4 for TCG and 6 for HEG). Home-practice adherence was calculated as completed home-practice days divided by 50 prescribed days during weeks 3–12. The two adherence components were summarized separately and were not averaged into an overall score. A threshold of at least 80% was summarized for each component.

The prescribed Tai Chi intensity target was 3–5 on the Borg CR10 scale. Session-level attainment of the intensity target is not reported because the previously summarized denominators could not be reliably traced to source records.

Nonserious adverse events were actively solicited during the 12-week intervention period, and serious adverse events were monitored through week 26. A masked study physician adjudicated seriousness and intervention relatedness using available clinical records. Symptom-relief treatments were summarized separately from adverse events.

Analyses were performed using R version 4.6.0.

# eResults. Additional Trial Results

## Follow-up Completeness and Missingness

Outcome assessments were complete through week 8. At week 12, TUG assessments were available for 41 of 48 participants in TCG and 40 of 48 in HEG; at week 26, they were available for 37 and 35 participants, respectively. All 15 participants who missed the week-12 assessment were successfully re-contacted by telephone and/or WeChat before week 26 and invited to return, but all declined further in-person assessment. Missingness was monotone for the primary outcome between weeks 12 and 26 (eTables 1 and 7).

## Responder Analyses

At week 12, the adjusted risk ratio for achieving the prespecified TUG responder threshold was 4.81 (95% CI, 2.29–10.07), and at week 26 it was 2.12 (95% CI, 1.07–4.20). Adjusted responder effects for KVAS and WOMAC total score are reported in eTable 3.

## Adherence, Dose, and Safety

Supervised-session adherence was 95.3% in TCG and 94.4% in HEG; 81.3% and 97.9%, respectively, met the at-least-80% supervised-session threshold. Home-practice adherence was 86.2% in TCG and 79.4% in HEG; 75.0% and 47.9%, respectively, completed at least 80% of prescribed home-practice days (eTable 4). Intervention dose and progression are summarized in eTable 5. Safety and symptom-relief treatment details are summarized in eTable 6.

## Sensitivity and Subgroup Analyses

Primary TUG estimates were similar in complete-case and MAR multiple-imputation analyses. Under the most unfavorable differential MNAR scenario examined (TCG +1.5 seconds; HEG +0 seconds), the adjusted between-group differences remained −2.32 seconds (95% CI, −3.22 to −1.41) at week 12 and −1.12 seconds (95% CI, −2.07 to −0.17) at week 26 (eTable 9).

The alternative categorical-time random-slope model yielded adjusted between-group differences of −2.51 seconds at week 12 and −1.39 seconds at week 26. The SAP-defined per-protocol analysis favored TCG at week 12 (−2.27 seconds; 95% CI, −3.69 to −0.86) but not clearly at week 26 (−0.68 seconds; 95% CI, −2.09 to 0.73) (eTable 10). Prespecified subgroup interaction p values were 0.292 for sex, 0.922 for age, 0.334 for BMI, and 0.370 for KL grade (eTable 11; eFigure 3).

# eTables

**eTable 1. Participant Completion by Visit, Randomization Group, and Study Site**

| **Visit** | **Group** | **XS, No./24 (%)** | **YY, No./24 (%)** | **Overall, No./48 (%)** |
| --- | --- | --- | --- | --- |
| Baseline | TCG | 24/24 (100.0) | 24/24 (100.0) | 48/48 (100.0) |
| Baseline | HEG | 24/24 (100.0) | 24/24 (100.0) | 48/48 (100.0) |
| Week 4 | TCG | 24/24 (100.0) | 24/24 (100.0) | 48/48 (100.0) |
| Week 4 | HEG | 24/24 (100.0) | 24/24 (100.0) | 48/48 (100.0) |
| Week 8 | TCG | 24/24 (100.0) | 24/24 (100.0) | 48/48 (100.0) |
| Week 8 | HEG | 24/24 (100.0) | 24/24 (100.0) | 48/48 (100.0) |
| Week 12 | TCG | 20/24 (83.3) | 21/24 (87.5) | 41/48 (85.4) |
| Week 12 | HEG | 20/24 (83.3) | 20/24 (83.3) | 40/48 (83.3) |
| Week 26 | TCG | 20/24 (83.3) | 17/24 (70.8) | 37/48 (77.1) |
| Week 26 | HEG | 17/24 (70.8) | 18/24 (75.0) | 35/48 (72.9) |

Completion was defined by availability of the TUG assessment at the specified visit. XS and YY each enrolled 48 participants, with 24 participants per randomized group at each site.

Abbreviations: HEG, health education group; TCG, Tai Chi group; TUG, Timed Up and Go.

**eTable 2. Full Adjusted Continuous Outcome Results Across All Follow-up Visits**

| **Outcome** | **Visit** | **TCG, adjusted change from baseline (95% CI)** | **HEG, adjusted change from baseline (95% CI)** | **Adjusted between-group difference in change (95% CI)** | **Standardized effect size, Cohen d (95% CI)** | **p-value** |
| --- | --- | --- | --- | --- | --- | --- |
| Timed Up and Go test, s | Week 4 | −0.92 (−1.53 to −0.31) | −0.79 (−1.39 to −0.18) | −0.14 (−1.00 to 0.73) | −0.06 (−0.46 to 0.33) | 0.757 |
| Timed Up and Go test, s | Week 8 | −1.75 (−2.36 to −1.14) | −1.39 (−2.00 to −0.78) | −0.36 (−1.22 to 0.50) | −0.17 (−0.56 to 0.23) | 0.414 |
| Timed Up and Go test, s | Week 12 | −3.36 (−4.00 to −2.72) | −0.84 (−1.48 to −0.19) | −2.52 (−3.43 to −1.61) | −1.16 (−1.58 to −0.74) | <0.001 |
| Timed Up and Go test, s | Week 26 | −3.01 (−3.67 to −2.35) | −1.55 (−2.22 to −0.87) | −1.46 (−2.41 to −0.52) | −0.67 (−1.11 to −0.24) | 0.002 |
| Knee Visual Analog Scale, mm | Week 4 | −5.60 (−7.95 to −3.26) | −7.56 (−9.90 to −5.22) | 1.96 (−1.35 to 5.27) | 0.30 (−0.20 to 0.80) | 0.246 |
| Knee Visual Analog Scale, mm | Week 8 | −8.81 (−11.15 to −6.47) | −9.52 (−11.86 to −7.18) | 0.71 (−2.60 to 4.02) | 0.11 (−0.39 to 0.61) | 0.674 |
| Knee Visual Analog Scale, mm | Week 12 | −23.28 (−25.73 to −20.84) | −20.47 (−22.93 to −18.01) | −2.82 (−6.28 to 0.65) | −0.43 (−0.95 to 0.10) | 0.111 |
| Knee Visual Analog Scale, mm | Week 26 | −19.06 (−21.57 to −16.54) | −18.25 (−20.81 to −15.69) | −0.81 (−4.39 to 2.78) | −0.12 (−0.66 to 0.42) | 0.659 |
| WOMAC total score, points | Week 4 | −9.25 (−13.88 to −4.62) | −13.17 (−17.80 to −8.54) | 3.92 (−2.63 to 10.47) | 0.41 (−0.27 to 1.08) | 0.240 |
| WOMAC total score, points | Week 8 | −31.46 (−36.09 to −26.83) | −31.52 (−36.15 to −26.89) | 0.06 (−6.49 to 6.61) | 0.01 (−0.67 to 0.68) | 0.985 |
| WOMAC total score, points | Week 12 | −54.03 (−58.86 to −49.20) | −47.23 (−52.09 to −42.37) | −6.80 (−13.65 to 0.05) | −0.70 (−1.41 to 0.01) | 0.052 |
| WOMAC total score, points | Week 26 | −43.69 (−48.66 to −38.72) | −34.39 (−39.44 to −29.34) | −9.30 (−16.38 to −2.21) | −0.96 (−1.70 to −0.23) | 0.010 |
| WOMAC pain, points | Week 4 | −4.69 (−6.08 to −3.29) | −5.25 (−6.65 to −3.85) | 0.56 (−1.41 to 2.54) | 0.15 (−0.39 to 0.69) | 0.576 |
| WOMAC pain, points | Week 8 | −10.52 (−11.92 to −9.12) | −10.73 (−12.13 to −9.33) | 0.21 (−1.77 to 2.18) | 0.06 (−0.48 to 0.60) | 0.836 |
| WOMAC pain, points | Week 12 | −21.09 (−22.54 to −19.63) | −17.86 (−19.33 to −16.40) | −3.22 (−5.29 to −1.16) | −0.88 (−1.45 to −0.32) | 0.002 |
| WOMAC pain, points | Week 26 | −23.61 (−25.11 to −22.11) | −19.90 (−21.43 to −18.38) | −3.71 (−5.84 to −1.57) | −1.01 (−1.60 to −0.43) | <0.001 |

Part A includes TUG, KVAS, WOMAC total score, and WOMAC pain across weeks 4, 8, 12, and 26.

**eTable 2. Full Adjusted Continuous Outcome Results Across All Follow-up Visits (continued)**

| **Outcome** | **Visit** | **TCG, adjusted change from baseline (95% CI)** | **HEG, adjusted change from baseline (95% CI)** | **Adjusted between-group difference in change (95% CI)** | **Standardized effect size, Cohen d (95% CI)** | **p-value** |
| --- | --- | --- | --- | --- | --- | --- |
| WOMAC stiffness, points | Week 4 | −2.96 (−3.61 to −2.30) | −3.12 (−3.78 to −2.47) | 0.17 (−0.76 to 1.09) | 0.07 (−0.31 to 0.45) | 0.723 |
| WOMAC stiffness, points | Week 8 | −4.79 (−5.45 to −4.14) | −4.52 (−5.17 to −3.87) | −0.27 (−1.20 to 0.65) | −0.11 (−0.49 to 0.27) | 0.565 |
| WOMAC stiffness, points | Week 12 | −8.22 (−8.90 to −7.54) | −8.13 (−8.81 to −7.44) | −0.09 (−1.06 to 0.88) | −0.04 (−0.44 to 0.36) | 0.855 |
| WOMAC stiffness, points | Week 26 | −8.04 (−8.74 to −7.33) | −7.36 (−8.07 to −6.65) | −0.68 (−1.68 to 0.32) | −0.28 (−0.69 to 0.13) | 0.184 |
| WOMAC physical function, points | Week 4 | −1.60 (−6.03 to 2.83) | −4.79 (−9.22 to −0.36) | 3.19 (−3.08 to 9.45) | 0.33 (−0.32 to 0.99) | 0.318 |
| WOMAC physical function, points | Week 8 | −16.15 (−20.58 to −11.72) | −16.27 (−20.70 to −11.84) | 0.13 (−6.14 to 6.39) | 0.01 (−0.64 to 0.67) | 0.969 |
| WOMAC physical function, points | Week 12 | −24.73 (−29.35 to −20.11) | −21.24 (−25.89 to −16.59) | −3.49 (−10.04 to 3.07) | −0.36 (−1.05 to 0.32) | 0.296 |
| WOMAC physical function, points | Week 26 | −12.04 (−16.79 to −7.29) | −7.13 (−11.96 to −2.29) | −4.91 (−11.69 to 1.86) | −0.51 (−1.22 to 0.19) | 0.155 |
| Grip strength, kg | Week 4 | −1.86 (−2.28 to −1.44) | −2.47 (−2.89 to −2.05) | 0.61 (0.02 to 1.21) | 0.29 (0.01 to 0.57) | 0.044 |
| Grip strength, kg | Week 8 | 0.21 (−0.21 to 0.63) | 0.64 (0.22 to 1.06) | −0.43 (−1.03 to 0.16) | −0.20 (−0.49 to 0.08) | 0.155 |
| Grip strength, kg | Week 12 | 1.90 (1.46 to 2.34) | 1.73 (1.28 to 2.18) | 0.17 (−0.46 to 0.80) | 0.08 (−0.22 to 0.38) | 0.599 |
| Grip strength, kg | Week 26 | 2.89 (2.43 to 3.35) | 2.72 (2.25 to 3.19) | 0.17 (−0.48 to 0.83) | 0.08 (−0.23 to 0.39) | 0.606 |
| Modified Falls Efficacy Scale, points | Week 4 | 5.42 (4.38 to 6.45) | 7.79 (6.76 to 8.83) | −2.38 (−3.84 to −0.91) | −0.22 (−0.35 to −0.08) | 0.002 |
| Modified Falls Efficacy Scale, points | Week 8 | 14.42 (13.38 to 15.45) | 13.79 (12.76 to 14.83) | 0.62 (−0.84 to 2.09) | 0.06 (−0.08 to 0.19) | 0.401 |
| Modified Falls Efficacy Scale, points | Week 12 | 26.19 (25.09 to 27.28) | 24.47 (23.37 to 25.57) | 1.72 (0.17 to 3.27) | 0.16 (0.02 to 0.30) | 0.030 |
| Modified Falls Efficacy Scale, points | Week 26 | 23.72 (22.59 to 24.85) | 21.37 (20.22 to 22.52) | 2.35 (0.73 to 3.96) | 0.21 (0.07 to 0.36) | 0.004 |
| Appendicular skeletal muscle mass index, kg/m² | Week 12 | 0.418 (0.357 to 0.479) | 0.508 (0.446 to 0.569) | −0.089 (−0.176 to −0.003) | −0.06 (−0.12 to 0.00) | 0.043 |

The same site-adjusted categorical-time linear mixed-effects models and covariance-aware contrasts used for main Table 2 were used here.

Cohen d was calculated by dividing the adjusted between-group difference in change by the pooled baseline SD for the corresponding outcome; confidence limits were standardized using the same pooled baseline SD.

Negative values favor TCG for TUG, KVAS, and WOMAC outcomes; positive values favor TCG for grip strength, MFES, and ASMI. ASMI was assessed at baseline and week 12 only.

No multiplicity adjustment was applied to secondary outcomes or nonprimary time points.

Abbreviations: ASMI, appendicular skeletal muscle mass index; CI, confidence interval; HEG, health education group; KVAS, Knee Visual Analog Scale; MFES, Modified Falls Efficacy Scale; TCG, Tai Chi group; TUG, Timed Up and Go; WOMAC, Western Ontario and McMaster Universities Osteoarthritis Index.

**eTable 3. Minimal Clinically Important Difference Responder Analyses at Weeks 12 and 26**

| **Outcome** | **Visit** | **MCID criterion** | **TCG responders, No. (%)** | **HEG responders, No. (%)** | **Unadjusted risk difference, % (95% CI)** | **Unadjusted RR (95% CI)** | **Adjusted RR (95% CI)** | **p-value** |
| --- | --- | --- | --- | --- | --- | --- | --- | --- |
| Timed Up and Go test, s | Week 12 | Reduction ≥ 2.8 s | 28/48 (58.3) | 6/48 (12.5) | 45.8 (27.2 to 60.3) | 4.67 (2.13 to 10.24) | 4.81 (2.29 to 10.07) | <0.001 |
| Timed Up and Go test, s | Week 26 | Reduction ≥ 2.8 s | 17/48 (35.4) | 8/48 (16.7) | 18.8 (1.1 to 35.0) | 2.13 (1.01 to 4.45) | 2.12 (1.07 to 4.20) | 0.031 |
| Knee Visual Analog Scale, mm | Week 12 | Reduction ≥ 15 mm | 32/48 (66.7) | 33/48 (68.8) | −2.1 (−20.2 to 16.2) | 0.97 (0.74 to 1.28) | 0.94 (0.71 to 1.24) | 0.657 |
| Knee Visual Analog Scale, mm | Week 26 | Reduction ≥ 15 mm | 29/48 (60.4) | 26/48 (54.2) | 6.2 (−13.1 to 25.0) | 1.12 (0.79 to 1.58) | 1.09 (0.76 to 1.56) | 0.648 |
| WOMAC total score, points | Week 12 | Reduction ≥ 40 points | 37/48 (77.1) | 30/48 (62.5) | 14.6 (−3.8 to 31.7) | 1.23 (0.94 to 1.61) | 1.24 (0.94 to 1.62) | 0.124 |
| WOMAC total score, points | Week 26 | Reduction ≥ 40 points | 25/48 (52.1) | 13/48 (27.1) | 25.0 (5.4 to 42.1) | 1.92 (1.12 to 3.29) | 1.92 (1.11 to 3.32) | 0.019 |

Responder status was defined using the prespecified MCID thresholds. Participants with missing follow-up data were classified as nonresponders, and denominators include all randomized participants (48 per group).

Risk difference is TCG minus HEG; positive values favor TCG. Unadjusted risk-difference confidence intervals use the Newcombe–Wilson method.

Adjusted risk ratios were estimated using modified Poisson regression with heteroskedasticity-robust standard errors, adjusting for study site, age, sex, body mass index, and Kellgren–Lawrence grade. RR >1 favors TCG.

No multiplicity adjustment was applied; responder analyses are exploratory.

Abbreviations: CI, confidence interval; HEG, health education group; MCID, minimal clinically important difference; RR, risk ratio; TCG, Tai Chi group.

**eTable 4. Adherence to Supervised Sessions and Home Practice During the Intervention**

## Panel A. Supervised-session adherence

| **Measure** | **TCG (n=48)** | **HEG (n=48)** |
| --- | --- | --- |
| Prescribed supervised sessions, No. | 4 | 6 |
| Sessions attended, mean (SD), No. | 3.81 (0.39) | 5.67 (0.52) |
| Supervised-session adherence, mean (SD), % | 95.3 (9.9) | 94.4 (8.7) |
| Participants with ≥80% supervised-session adherence, No. (%) | 39 (81.3%) | 47 (97.9%) |

## Panel B. Home-practice adherence

| **Measure** | **TCG (n=48)** | **HEG (n=48)** |
| --- | --- | --- |
| Prescribed home-practice days, No. | 50 | 50 |
| Home-practice days completed, mean (SD), No. | 43.10 (4.26) | 39.71 (4.59) |
| Home-practice adherence, mean (SD), % | 86.2 (8.5) | 79.4 (9.2) |
| Participants with ≥80% home-practice adherence, No. (%) | 36 (75.0%) | 23 (47.9%) |

Supervised-session adherence was calculated as sessions attended divided by prescribed supervised sessions (4 for TCG and 6 for HEG).

Home-practice adherence was calculated as completed home-practice days divided by 50 prescribed days (5 days/week during weeks 3–12).

The ≥80% threshold corresponds to 4/4 supervised sessions in TCG, at least 5/6 supervised sessions in HEG, and at least 40/50 home-practice days in both groups. Supervised and home adherence were not averaged into an overall adherence score.

Abbreviations: HEG, health education group; TCG, Tai Chi group.

**eTable 5. Intervention Dose and Progression During the 12-Week Intervention**

| **Measure** | **TCG (n=48)** | **HEG (n=48)** |
| --- | --- | --- |
| Prescribed supervised sessions, No. | 4 | 6 |
| Prescribed duration per supervised session, min | 60 | 60 |
| Prescribed home-practice frequency, days/week | 5 | 5 |
| Prescribed home-practice period, weeks | 3–12 | 3–12 |
| Prescribed home-practice duration, min/day | 30 | 20 |
| Observed home-practice session duration, mean (SD), min | 29.4 (5.8) | 24.7 (6.1) |
| Participants completing the planned final intervention phase, No. (%) | 42 (87.5%) | 45 (93.8%) |

Observed home-practice session duration was derived from weekly WeChat/home-practice logs.

The target perceived exertion was 3–5 on the Borg CR10 scale; no session-level RPE attainment proportion is reported because the previously summarized denominators could not be reliably traced to source records.

The final planned intervention phase refers to weeks 9–12 of the group-specific progression. No movement-accuracy or video-fidelity results are reported because source-verifiable participant-level data were unavailable.

Abbreviations: HEG, health education group; TCG, Tai Chi group.

**eTable 6. Adverse Events and Symptom-Relief Treatments**

## Panel A. Adverse events

| **Event** | **Severity** | **Relatedness** | **Time window** | **TCG (n=48), No. (%)** | **HEG (n=48), No. (%)** |
| --- | --- | --- | --- | --- | --- |
| Participants with ≥1 nonserious AE | Nonserious | - | Weeks 0–12 | 6 (12.5) | 3 (6.3) |
| Transient knee pain exacerbation | Mild | Possibly related | Weeks 0–12 | 4 (8.3) | 0 |
| Ankle sprain | Mild | Possibly related | Weeks 0–12 | 2 (4.2) | 0 |
| Thigh muscle soreness | Mild | Possibly related | Weeks 0–12 | 0 | 3 (6.3) |
| Participants with ≥1 SAE | Serious | Unrelated/unlikely related | Through week 26 | 3 (6.3) | 2 (4.2) |
| Cardiovascular hospitalization | Serious | Unrelated/unlikely related | Weeks 0–12 | 2 (4.2) | 1 (2.1) |
| Cardiovascular hospitalization | Serious | Unrelated/unlikely related | Weeks 12–26 | 1 (2.1) | 1 (2.1) |
| Withdrawal because of intervention-related AE | - | - | Through week 26 | 0 | 0 |

## Panel B. Symptom-relief treatments

| **Symptom-relief treatment (weeks 0–12)** | **TCG (n=48), No. (%)** | **HEG (n=48), No. (%)** |
| --- | --- | --- |
| Participants using ≥1 symptom-relief treatment | 6 (12.5) | 3 (6.3) |
| Etoricoxib | 6 (12.5) | 3 (6.3) |
| Topical herbal heat compress | 0 | 3 (6.3) |
| No symptom-relief treatment | 42 (87.5) | 45 (93.8) |

Participants could report more than one adverse event. Nonserious musculoskeletal events were considered possibly related to study participation because of their temporal association with intervention sessions or practice.

Serious adverse events were monitored through week 26. All SAEs were cardiovascular hospitalizations and were judged unrelated or unlikely related to the study interventions by the masked study physician using available clinical records.

Symptom-relief treatments were recorded separately from adverse events. Treatment categories were not mutually exclusive. In HEG, the same 3 participants who used etoricoxib also used topical herbal heat compresses.

Abbreviations: AE, adverse event; HEG, health education group; SAE, serious adverse event; TCG, Tai Chi group.

**eTable 7. Follow-up Completeness and Missing-Data Patterns by Randomization Group**

## Panel A. Outcome assessment completeness by visit

| **Visit** | **TCG complete, No./48 (%)** | **HEG complete, No./48 (%)** | **Total complete, No./96 (%)** |
| --- | --- | --- | --- |
| Baseline | 48/48 (100.0) | 48/48 (100.0) | 96/96 (100.0) |
| Week 4 | 48/48 (100.0) | 48/48 (100.0) | 96/96 (100.0) |
| Week 8 | 48/48 (100.0) | 48/48 (100.0) | 96/96 (100.0) |
| Week 12 | 41/48 (85.4) | 40/48 (83.3) | 81/96 (84.4) |
| Week 26 | 37/48 (77.1) | 35/48 (72.9) | 72/96 (75.0) |

## Panel B. Missing-data patterns at weeks 12 and 26

| **Missing-data pattern** | **TCG (n=48), No. (%)** | **HEG (n=48), No. (%)** |
| --- | --- | --- |
| Week 12 and week 26 both completed | 37 (77.1%) | 35 (72.9%) |
| Week 12 completed; week 26 missing | 4 (8.3%) | 5 (10.4%) |
| Week 12 and week 26 both missing | 7 (14.6%) | 8 (16.7%) |
| Week 12 missing; week 26 completed | 0 (0.0%) | 0 (0.0%) |

Completeness was defined by availability of the TUG assessment at the specified visit. Missingness of the key continuous outcomes was aligned within each follow-up visit.

All 15 participants who did not complete the week-12 assessment were re-contacted by telephone and/or WeChat before week 26 and invited to return for outcome assessment; all declined to return for further in-person assessment, and none completed the week-26 assessment.

Raw missing values remained missing in the analysis data. Missing-data assumptions and sensitivity analyses are reported separately in eTable 9.

Abbreviations: HEG, health education group; TCG, Tai Chi group; TUG, Timed Up and Go.

**eTable 8. Baseline Characteristics by Completeness of Outcome Assessment**

## Panel A. Week 12 outcome assessment

| **Characteristic** | **Complete (n=81)** | **Incomplete (n=15)** |
| --- | --- | --- |
| Age, y | 72.3 (3.2) | 72.1 (3.0) |
| Female, No. (%) | 29 (35.8%) | 5 (33.3%) |
| Study site, No. (%) |  |  |
| XS | 40 (49.4%) | 8 (53.3%) |
| YY | 41 (50.6%) | 7 (46.7%) |
| BMI, kg/m² | 22.2 (3.3) | 22.2 (2.3) |
| Kellgren–Lawrence grade, No. (%) |  |  |
| Grade I | 7 (8.6%) | 4 (26.7%) |
| Grade II | 36 (44.4%) | 8 (53.3%) |
| Grade III | 31 (38.3%) | 2 (13.3%) |
| Grade IV | 7 (8.6%) | 1 (6.7%) |
| Baseline TUG, s | 13.2 (2.3) | 13.2 (1.6) |
| Baseline KVAS, mm | 60.1 (6.7) | 60.1 (6.5) |
| Baseline WOMAC total score, points | 150.3 (9.6) | 143.5 (7.7) |

## Panel B. Week 26 outcome assessment

| **Characteristic** | **Complete (n=72)** | **Incomplete (n=24)** |
| --- | --- | --- |
| Age, y | 72.2 (3.1) | 72.3 (3.3) |
| Female, No. (%) | 26 (36.1%) | 8 (33.3%) |
| Study site, No. (%) |  |  |
| XS | 37 (51.4%) | 11 (45.8%) |
| YY | 35 (48.6%) | 13 (54.2%) |
| BMI, kg/m² | 22.0 (3.1) | 22.9 (3.2) |
| Kellgren–Lawrence grade, No. (%) |  |  |
| Grade I | 6 (8.3%) | 5 (20.8%) |
| Grade II | 32 (44.4%) | 12 (50.0%) |
| Grade III | 27 (37.5%) | 6 (25.0%) |
| Grade IV | 7 (9.7%) | 1 (4.2%) |
| Baseline TUG, s | 13.2 (2.2) | 13.3 (1.9) |
| Baseline KVAS, mm | 60.0 (6.1) | 60.4 (8.3) |
| Baseline WOMAC total score, points | 150.9 (9.8) | 144.0 (6.9) |

Completeness was defined according to availability of the primary outcome, Timed Up and Go test, at the respective follow-up visit.

Continuous variables are presented as mean (SD) and categorical variables as No. (%). These comparisons are descriptive; no formal statistical tests or p values were calculated.

Abbreviations: BMI, body mass index; KVAS, Knee Visual Analog Scale; TUG, Timed Up and Go; WOMAC, Western Ontario and McMaster Universities Osteoarthritis Index; XS, Xiangshan site; YY, Yuyuan site.

**eTable 9. Sensitivity Analyses for Missing Outcome Data for the Timed Up and Go Test**

## Panel A. Primary and MAR-based sensitivity analyses

| **Analysis** | **Missing-data assumption** | **Visit** | **Participants in analysis, No.** | **Adjusted between-group difference in change, s (95% CI)** | **p-value** |
| --- | --- | --- | --- | --- | --- |
| Primary LMM | MAR | Week 12 | 96 | −2.52 (−3.43 to −1.61) | <0.001 |
| Primary LMM | MAR | Week 26 | 96 | −1.46 (−2.41 to −0.52) | 0.002 |
| Complete-case analysis | Observed target-visit cases | Week 12 | 81 | −2.51 (−3.47 to −1.56) | <0.001 |
| Complete-case analysis | Observed target-visit cases | Week 26 | 72 | −1.54 (−2.58 to −0.51) | 0.004 |
| Multiple imputation | MAR | Week 12 | 96 | −2.54 (−3.43 to −1.64) | <0.001 |
| Multiple imputation | MAR | Week 26 | 96 | −1.46 (−2.41 to −0.52) | 0.002 |

## Panel B. MNAR delta-adjusted pattern-mixture sensitivity analyses

| **MNAR scenario** | **Delta applied to missing TUG** | **Visit** | **Adjusted between-group difference in change, s (95% CI)** | **p-value** |
| --- | --- | --- | --- | --- |
| Equal penalty | TCG +0.5 s; HEG +0.5 s | Week 12 | −2.55 (−3.44 to −1.65) | <0.001 |
| Equal penalty | TCG +0.5 s; HEG +0.5 s | Week 26 | −1.48 (−2.43 to −0.54) | 0.002 |
| Equal penalty | TCG +1.0 s; HEG +1.0 s | Week 12 | −2.56 (−3.46 to −1.65) | <0.001 |
| Equal penalty | TCG +1.0 s; HEG +1.0 s | Week 26 | −1.50 (−2.46 to −0.55) | 0.002 |
| Equal penalty | TCG +1.5 s; HEG +1.5 s | Week 12 | −2.57 (−3.48 to −1.65) | <0.001 |
| Equal penalty | TCG +1.5 s; HEG +1.5 s | Week 26 | −1.52 (−2.49 to −0.56) | 0.002 |
| Differential penalty | TCG +0.5 s; HEG +0 s | Week 12 | −2.46 (−3.36 to −1.57) | <0.001 |
| Differential penalty | TCG +0.5 s; HEG +0 s | Week 26 | −1.35 (−2.29 to −0.40) | 0.005 |
| Differential penalty | TCG +1.0 s; HEG +0 s | Week 12 | −2.39 (−3.29 to −1.49) | <0.001 |
| Differential penalty | TCG +1.0 s; HEG +0 s | Week 26 | −1.23 (−2.18 to −0.29) | 0.011 |
| Differential penalty | TCG +1.5 s; HEG +0 s | Week 12 | −2.32 (−3.22 to −1.41) | <0.001 |
| Differential penalty | TCG +1.5 s; HEG +0 s | Week 26 | −1.12 (−2.07 to −0.17) | 0.021 |

Between-group differences represent (TCG follow-up − TCG baseline) − (HEG follow-up − HEG baseline); negative values favor TCG.

The primary LMM included treatment group, categorical visit, treatment-by-visit interaction, study site, age, sex, body mass index, and Kellgren–Lawrence grade, with a participant-specific random intercept. The likelihood-based primary analysis assumes data are MAR conditional on observed outcomes and covariates.

MAR multiple imputation used 20 imputed data sets generated using the mice 2l.pan method in long format with participant as the clustering variable. Treatment-by-visit terms and the analysis covariates were included in the imputation model. Estimates were combined using Rubin's rules.

For MNAR sensitivity analyses, positive delta values were added only to originally missing follow-up TUG values after MAR imputation; positive deltas represent worse TUG performance than predicted under MAR. Differential scenarios deliberately penalized TCG more strongly to test assumptions unfavorable to Tai Chi.

p values are 2-sided. Abbreviations: CI, confidence interval; HEG, health education group; LMM, linear mixed-effects model; MAR, missing at random; MI, multiple imputation; MNAR, missing not at random; TCG, Tai Chi group; TUG, Timed Up and Go.

**eTable 10. Additional Sensitivity Analyses for the Primary Outcome**

| **Analysis** | **Visit** | **Participants in analysis, No.** | **Analysis population / model** | **Adjusted between-group difference in change, s (95% CI)** | **p-value** |
| --- | --- | --- | --- | --- | --- |
| Primary analysis | Week 12 | 96 | ITT; categorical-time random-intercept LMM | −2.52 (−3.43 to −1.61) | <0.001 |
| Primary analysis | Week 26 | 96 | ITT; categorical-time random-intercept LMM | −1.46 (−2.41 to −0.52) | 0.002 |
| Alternative categorical-time random-slope model | Week 12 | 96 | Categorical fixed time; uncorrelated random intercept and numeric-time slope | −2.51 (−3.39 to −1.63) | <0.001 |
| Alternative categorical-time random-slope model | Week 26 | 96 | Categorical fixed time; uncorrelated random intercept and numeric-time slope | −1.39 (−2.54 to −0.24) | 0.018 |
| Outlier-removed analysis | Week 12 | 96 | Excluded 3 observation(s) with \|standardized residual\| > 3 | −2.65 (−3.50 to −1.80) | <0.001 |
| Outlier-removed analysis | Week 26 | 96 | Excluded 3 observation(s) with \|standardized residual\| > 3 | −1.25 (−2.15 to −0.36) | 0.006 |
| Per-protocol analysis | Week 12 | 40 | SAP V1.2 PP: ≥80% supervised + ≥80% home adherence + week-26 assessment | −2.27 (−3.69 to −0.86) | 0.002 |
| Per-protocol analysis | Week 26 | 40 | SAP V1.2 PP: ≥80% supervised + ≥80% home adherence + week-26 assessment | −0.68 (−2.09 to 0.73) | 0.344 |

Adjusted between-group differences represent (TCG follow-up − TCG baseline) − (HEG follow-up − HEG baseline); negative values favor TCG.

The primary analysis used an LMM including treatment group, categorical visit, treatment-by-visit interaction, study site, age, sex, body mass index, and Kellgren–Lawrence grade, with a participant-specific random intercept.

The alternative model retained categorical visit and the same fixed-effects adjustment as the primary model, while adding an uncorrelated participant-specific random slope for numeric time scaled in 4-week units. All available TUG measurements at baseline and weeks 4, 8, 12, and 26 were included.

For the outlier-removed analysis, observations with an absolute standardized conditional residual >3 in the primary model were excluded and the primary categorical-time model was refitted.

The PP analysis follows SAP version 1.2: participants had ≥80% supervised-session adherence, ≥80% home-practice adherence, and a week-26 assessment.

p values are 2-sided. These sensitivity analyses were not adjusted for multiple comparisons. Abbreviations: CI, confidence interval; HEG, health education group; ITT, intention to treat; LMM, linear mixed-effects model; PP, per protocol; TCG, Tai Chi group; TUG, Timed Up and Go.

**eTable 11. Prespecified Exploratory Subgroup Analyses of the Treatment Effect on TUG at Week 12**

| **Subgroup** | **Category** | **No. randomized** | **Adjusted between-group difference in change, s (95% CI)** | **p for interaction** |
| --- | --- | --- | --- | --- |
| Sex | Female | 34 | −3.19 (−4.71 to −1.66) | 0.292 |
|  | Male | 62 | −2.17 (−3.30 to −1.03) |  |
| Age | <75 y | 70 | −2.49 (−3.57 to −1.40) | 0.922 |
|  | ≥75 y | 26 | −2.59 (−4.30 to −0.88) |  |
| BMI | <24 kg/m² | 69 | −2.28 (−3.36 to −1.20) | 0.334 |
|  | ≥24 kg/m² | 27 | −3.26 (−4.95 to −1.57) |  |
| Kellgren–Lawrence grade | I–II | 55 | −2.10 (−3.34 to −0.85) | 0.370 |
|  | III–IV | 41 | −2.94 (−4.32 to −1.57) |  |

The prespecified exploratory subgroups were sex (female vs male), age (<75 vs ≥75 years), BMI (<24 vs ≥24 kg/m²), and Kellgren–Lawrence grade (I–II vs III–IV).

For each subgroup factor, an LMM included treatment group, categorical visit, subgroup, all corresponding 2-way and 3-way interaction terms, study site, the remaining prespecified baseline covariates, and a participant-specific random intercept. The baseline covariate defining a given subgroup was not additionally entered in its original form in that subgroup model.

Adjusted between-group differences represent (TCG week 12 − TCG baseline) − (HEG week 12 − HEG baseline); negative values favor TCG.

Interaction p values test whether the adjusted week-12 treatment effect differed between the two categories of each subgroup. No adjustment was made for multiple subgroup comparisons.

Abbreviations: BMI, body mass index; CI, confidence interval; HEG, health education group; KL, Kellgren–Lawrence; TCG, Tai Chi group; TUG, Timed Up and Go.

# eFigures

**eFigure 1. Schedule of Assessments Across the Trial Timeline**

Schedule of outcome assessments across the trial. TUG, KVAS, WOMAC, grip strength, and MFES were assessed at baseline and weeks 4, 8, 12, and 26; ASMI was assessed at baseline and week 12 only. The intervention period was weeks 0–12. Abbreviations: ASMI, appendicular skeletal muscle mass index; KVAS, Knee Visual Analog Scale; MFES, Modified Falls Efficacy Scale; TUG, Timed Up and Go; WOMAC, Western Ontario and McMaster Universities Osteoarthritis Index.

**eFigure 2. Model-Estimated Trajectories for Secondary Continuous Outcomes**

Model-estimated trajectories for (A) WOMAC stiffness, (B) WOMAC physical function, (C) grip strength, (D) Modified Falls Efficacy Scale, and (E) appendicular skeletal muscle mass index. Points and 95% CIs are estimated marginal means from the site-adjusted categorical-time mixed-effects models. ASMI was assessed at baseline and week 12 only.

**eFigure 3. Prespecified Exploratory Subgroup Analyses of Week-12 TUG Treatment Effect**

Adjusted between-group differences in TUG change from baseline to week 12 across prespecified exploratory subgroups. Negative values favor TCG. Interaction p values test treatment-effect heterogeneity. Models adjusted for study site and the remaining prespecified baseline covariates. Abbreviations: BMI, body mass index; CI, confidence interval; HEG, health education group; TCG, Tai Chi group; TUG, Timed Up and Go.

**eFigure 4. Delta-Adjusted MNAR Sensitivity Analysis for Missing TUG Outcomes**

Adjusted between-group differences in TUG change at weeks 12 and 26 across delta-adjusted pattern-mixture scenarios. Equal-penalty scenarios apply the same positive delta to missing TUG values in both groups; differential-penalty scenarios apply the delta only to missing TUG values in TCG, thereby imposing assumptions unfavorable to Tai Chi. Negative values favor TCG. Abbreviations: HEG, health education group; MNAR, missing not at random; TCG, Tai Chi group; TUG, Timed Up and Go.

# eAppendix. Intervention Details for the Tai Chi and Health Education Groups

This eAppendix summarizes the verified intervention content, schedule, progression, adherence monitoring, and key safety instructions used in the trial. It is intended to supplement the concise intervention description in the main manuscript.

## Tai Chi Group (TCG)

Participants received a standardized simplified 8-form Yang-style Tai Chi program adapted for older adults with knee pain and mobility limitations. The 8 forms were Commencement, Brush Knee and Step Forward, Play Guitar, Repulse Monkey, Single Whip, Cloud Hands, Part the Wild Horse's Mane, and Closing Form. Training emphasized upright alignment, controlled weight shifting, smooth transitional movements, and breath–movement coordination.

During weeks 1–2, participants attended 4 supervised 60-minute sessions (2 sessions/week). During weeks 3–12, home Tai Chi practice was prescribed 5 days/week for approximately 30 minutes/day. Practice progressed from full-sequence training to balance-focused consolidation. Moderate intensity was prescribed using a Borg CR10 target of 3–5. Participants were instructed to stop practice for concerning symptoms or if knee pain exceeded 3/10. Home practice was monitored using weekly participant-reported logs collected through WeChat, with periodic review of submitted practice videos.

**eAppendix Table 1. Tai Chi Intervention Schedule and Progression**

| **Weeks** | **Phase** | **Setting/content** | **Frequency** | **Dose / key focus** |
| --- | --- | --- | --- | --- |
| 1–2 | Instructional | Supervised teaching: movement decomposition, upright alignment, controlled weight shifting, breath–movement coordination, and correction of movement technique | 2 sessions/week (4 total) | 60 min/session |
| 3–8 | Home practice | Full 8-form sequence practice with emphasis on smooth transitions, knee alignment, and coordinated breathing | 5 days/week | ≈30 min/day; Borg CR10 target 3–5 |
| 9–12 | Consolidation | Continued 8-form practice with greater emphasis on balance control and stable transitions as tolerated | 5 days/week | ≈30 min/day; maintain safe, controlled movement |

Participants were instructed to stop for concerning symptoms or knee pain >3/10 and to avoid breath-holding or unsafe weight-bearing positions.

## Health Education Group (HEG)

Participants attended 6 structured 60-minute health education sessions delivered approximately every 2 weeks. Education covered SPKOA, dietary protein, fall prevention, and self-management. No home exercise was prescribed during weeks 1–2. During weeks 3–12, participants performed a progressive home program 5 days/week for approximately 20 minutes/day. The program incorporated lower-extremity strengthening, functional mobility, balance, stair practice, and later cognitive-motor tasks.

Participants were instructed to perform exercises near stable support, prioritize safe technique, and stop if knee pain exceeded 3/10. Progression was introduced only as tolerated. Home practice was monitored using weekly participant-reported logs collected through WeChat.

**eAppendix Table 2. Health Education and Home-Exercise Schedule**

| **Weeks** | **Component** | **Content** | **Frequency** | **Dose / progression** |
| --- | --- | --- | --- | --- |
| 1–12 | Health education | Structured education on SPKOA, dietary protein, fall prevention, and self-management | 6 sessions total, approximately every 2 weeks | 60 min/session |
| 1–2 | Home exercise | No prescribed home exercise | — | — |
| 3–8 | Home exercise | Progressive lower-extremity strengthening, functional mobility, and balance activities | 5 days/week | ≈20 min/day; progress as tolerated using stable support |
| 9–12 | Home exercise progression | Continued strengthening and balance with stair practice and later cognitive-motor tasks | 5 days/week | ≈20 min/day; reduce support/increase challenge only when safe |

The 6 supervised health education sessions were delivered over the 12-week intervention period, approximately every 2 weeks. No home exercise was prescribed during weeks 1–2.

## Abbreviations

AE, adverse event; ASMI, appendicular skeletal muscle mass index; BMI, body mass index; CI, confidence interval; HEG, health education group; ITT, intention to treat; KL, Kellgren–Lawrence; KVAS, Knee Visual Analog Scale; LMM, linear mixed-effects model; MAR, missing at random; MCID, minimal clinically important difference; MFES, Modified Falls Efficacy Scale; MI, multiple imputation; MNAR, missing not at random; PP, per protocol; RR, risk ratio; SAE, serious adverse event; TCG, Tai Chi group; TUG, Timed Up and Go; WOMAC, Western Ontario and McMaster Universities Osteoarthritis Index.
