# Supplementary material for: Effect of 8-form Tai Chi vs health education on mobility in older adults with sarcopenic knee osteoarthritis: A randomized clinical trial
Source: Osteoarthr Cartil Open. 2026 Sep 23;8(4):100881. doi: 10.1016/j.ocarto.2026.100881 (PMC13675917; doi:10.1016/j.ocarto.2026.100881)
Supplement: Supplementary file 2 [file mmc2.docx]

**Supplement 2. Trial Protocol**

**Protocol Title**

Eight-Form Tai Chi for Sarcopenic Knee Osteoarthritis (SPKOA): A 2-Arm, Assessor-Masked, Community-Based Randomized Clinical Trial

**Protocol Summary**

Design: Parallel-group, assessor-masked randomized clinical trial (1:1 allocation).

Setting: Community-based sites in Shanghai, China.

Population: Community-dwelling older adults with sarcopenic knee osteoarthritis (SPKOA).

Interventions: 12-week 8-form Yang-style Tai Chi program vs health education plus multicomponent home exercise.

Assessments: Baseline and weeks 4, 8, 12 (primary end point), and 26 (postintervention follow-up).

Primary outcome: Timed Up and Go (TUG) time (seconds) at week 12.

**1. Background and Rationale**

Knee osteoarthritis is a leading cause of pain and disability in older adults and commonly coexists with sarcopenia, a muscle disease characterized by low muscle strength and/or mass that increases vulnerability to functional decline. Sarcopenia and osteoarthritis may interact through reduced physical activity, pain-related movement adaptations, and deconditioning, potentially worsening mobility and quality of life.^1–5^

Exercise is recommended as first-line nonpharmacologic management for osteoarthritis and as a core intervention for sarcopenia.^6,7^ Tai Chi is a mind-body exercise that may improve mobility, balance, pain, and physical function in knee osteoarthritis and older adults.^8–13^ However, evidence specific to sarcopenic knee osteoarthritis remains limited. This trial evaluates an 8-form Yang-style Tai Chi program in community-dwelling older adults with SPKOA.

**2. Objectives and Hypotheses**

Primary objective: To estimate the adjusted between-group difference in change from baseline in TUG time at week 12 (Tai Chi guiding group [TCG] minus health education group [HEG]).

Secondary objectives: To compare groups on knee pain (KVAS), WOMAC total and subscales, grip strength, fall-related self-efficacy (MFES), and appendicular skeletal muscle mass index (ASMI), across follow-up time points.

Exploratory objective: To compare proportions achieving prespecified minimal clinically important difference (MCID) thresholds at weeks 12 and 26.

**3. Trial Design**

This is a 2-arm, parallel-group, assessor-masked randomized clinical trial with 1:1 allocation to TCG or HEG. Assessments occur at baseline and at weeks 4, 8, 12, and 26.

**4. Participants**

**4.1 Eligibility Criteria**

Eligible participants are community-dwelling older adults diagnosed with knee osteoarthritis and sarcopenia based on prespecified criteria. Radiographic severity is graded using the Kellgren–Lawrence (K–L) system.^14-15^ Key exclusion criteria include conditions that preclude safe participation in exercise or completion of outcome assessments.

**4.2 Recruitment and Consent**

Potential participants are prescreened and then undergo clinical screening and baseline assessment. Written informed consent is obtained prior to any study procedures, consistent with institutional requirements.

**4.3 Setting**

The trial is conducted in community-based settings in Shanghai, China.

**5. Randomization and Masking**

Randomization is performed using a computer-generated sequence with allocation concealment. Outcome assessors and data analysts are masked to group assignment. Participants and instructors are not masked because of the nature of the interventions.

**6. Interventions**

**6.1 Tai Chi Guiding Group (TCG)**

Participants receive a standardized 8-form Yang-style Tai Chi program over 12 weeks. The program includes 4 supervised instruction sessions delivered twice weekly during weeks 1–2, followed by structured home practice during weeks 3–12. Intensity is monitored using the Borg Category-Ratio 10 (CR10) scale, targeting moderate intensity (CR10 ≥5) during practice. Participants are instructed on safe technique (eg, knee alignment, controlled weight shifting) and to stop practice if pain increases substantially.

**6.2 Health Education Group (HEG)**

Participants receive 12 weeks of health education delivered in 6 supervised group sessions (approximately once every 2 weeks) and a multicomponent home exercise program prescribed for 20 minutes per day, 5 days per week during weeks 3–12. Exercises are progressed as tolerated with emphasis on safety and correct technique.

**Table 1. Tai Chi intervention schedule and progression (Weeks 1–12)**

| Weeks | Phase | Content | Frequency | Duration | Key focus |
| --- | --- | --- | --- | --- | --- |
| 1–2 | Instructional | Supervised teaching (movement decomposition, postural alignment, breath–movement coordination); video demonstration; real-time correction | 2 sessions/week (4 supervised sessions total) | 30 min (≈5-min warm-up, 20-min practice, 5-min cool-down) | ≥80% movement accuracy; correct knee alignment and weight distribution |
| 3–8 | Intensive practice | Home practice: full 8-form sequence ×3 rounds; 5-min warm-up; 5-min rest between rounds | 5 days/week | ≈30 min/day | Synchronize breath with movement; avoid breath-holding; smooth weight shifting |
| 9–12 | Consolidation | Home practice: fluid sequence ×3 rounds; add dynamic balance challenges (eg, transitional single-leg stances) as tolerated | 5 days/week | ≈30 min/day | Upright posture; controlled turns; minimize excessive center-of-mass displacement |

Notes: Warm-up and cool-down may include gentle joint mobility and breathing exercises.

**Table 2. Health education group home exercise progression (Weeks 1–12)**

| Weeks | Phase | Exercises / activities | Frequency | Dose | Progression / notes |
| --- | --- | --- | --- | --- | --- |
| 1–2 | Familiarization | Ankle dorsiflexion/plantarflexion; straight leg raise (supine); quadriceps sets; sit-to-stand | 5 days/week | 10 reps ×3 sets (exercises 1–3); 5 reps ×2 sets (sit-to-stand) | Use armrests initially; progress to no hands as tolerated |
| 3–8 | Basic training | Continue above exercises; emphasize technique and regularity | 5 days/week | As above | Adjust range of motion and speed based on pain and stability |
| 9–12 | Advanced training | Elastic-band straight leg raise; single-leg stance (eyes open→eyes closed); marching in place; stair negotiation practice | 5 days/week | 8 reps ×3 sets (resistance); 30 sec ×2 (balance) | Increase resistance; reduce support; add dual-task only if safe |

Abbreviations: HEG, health education group; TCG, Tai Chi guiding group.

**7. Outcomes and Assessments**

**7.1 Primary Outcome**

Timed Up and Go (TUG; seconds).^16-17^

**7.2 Secondary Outcomes**

Knee pain intensity by Knee Visual Analog Scale (KVAS; 0–100 mm).21,22 WOMAC total score and subscales (pain, stiffness, physical function).23 Grip strength (kg), Modified Falls Efficacy Scale (MFES; points), and ASMI (kg/m²; baseline and week 12 only).

**7.3 Responder Outcomes (MCID)**

Responder status is defined using prespecified MCID thresholds: TUG improvement ≥2.8 seconds, KVAS reduction ≥15 mm, and WOMAC total score reduction ≥40 points.^18-20^ Participants with missing follow-up data are classified as nonresponders for responder analyses.

**8. Sample Size**

The trial enrolls 96 participants (48 per group). The sample size was selected to provide adequate precision for the primary estimand based on expected between-group differences in mobility outcomes and feasibility in community settings.

**9. Data Collection and Management**

Data are collected at each study visit using standardized procedures. Outcome assessors receive training and use prespecified measurement protocols. Data entry and cleaning procedures are conducted prior to analysis, with masking maintained for analysts until the database is locked.

**10. Harms and Safety Monitoring**

Adverse events (AEs) are collected throughout the intervention and follow-up using structured queries at study visits and participant self-report. Events are classified as nonserious or serious (eg, requiring hospitalization) and graded by severity (mild, moderate, severe) when applicable. Relatedness to study participation is assessed by investigators (possibly related vs unrelated/unlikely related).

**11. Statistical Analysis Plan (Overview)**

Analyses follow the intention-to-treat principle. For continuous outcomes, linear mixed-effects models are used with fixed effects for group, time (categorical), and group×time interaction, adjusted for age, sex, body mass index, and K–L grade, with participant-specific random intercepts. Model-based contrasts estimate within-group changes from baseline and between-group differences in change from baseline (difference-in-differences) at each time point.

The primary hypothesis test is prespecified for TUG at week 12 (two-sided α=0.05). Secondary outcomes and other time points are exploratory. Missing data are handled using maximum likelihood under a missing-at-random assumption; sensitivity analyses include complete-case restrictions, alternative random-effects structures, outlier influence checks, and multiple imputation using a 2-level joint-model approach (2l.pan) with 20 imputations.

Responder analyses summarize group-specific proportions achieving MCID at weeks 12 and 26, with risk differences (percentage points) and risk ratios with 95% confidence intervals.

**12. Ethics and Dissemination**

The study is conducted in accordance with the Declaration of Helsinki and relevant local regulations. All participants provide written informed consent. Results will be disseminated through peer-reviewed publication and presentations.

**References**

1. Cruz-Jentoft AJ, Sayer AA. Sarcopenia. The Lancet. 2019;393(10191):2636-2646. doi:10.1016/S0140-6736(19)31138-9

2. Hunter DJ, Bierma-Zeinstra S. Osteoarthritis. The Lancet. 2019;393(10182):1745-1759. doi:10.1016/S0140-6736(19)30417-9

3. Chen LK, Liu LK, Woo J, et al. Sarcopenia in Asia: consensus report of the asian working group for sarcopenia. J Am Med Dir Assoc. 2014;15(2):95-101. doi:10.1016/j.jamda.2013.11.025

4. Chen TX, Yang TJ, Cheng S, Zhang ZL, Liu T. High prevalence and multifactorial risks of sarcopenia in knee osteoarthritis: A systematic review and meta-analysis. J Orthop Surg. 2025;20(1):987. doi:10.1186/s13018-025-06391-x

5. Liu GM, Sun B, Yang JY, et al. Development and validation of the BPDKC model for predicting sarcopenic knee osteoarthritis in community-dwelling older adults: A cross-sectional study. BMC Musculoskelet Disord. 2025;26(1):1072. doi:10.1186/s12891-025-09311-6

6. Bannuru RR, Osani MC, Vaysbrot EE, et al. OARSI guidelines for the non-surgical management of knee, hip, and polyarticular osteoarthritis. Osteoarthritis Cartilage. 2019;27(11):1578-1589. doi:10.1016/j.joca.2019.06.011

7. Shen Y, Shi Q, Nong K, et al. Exercise for sarcopenia in older people: a systematic review and network meta-analysis. J Cachexia Sarcopenia Muscle. 2023;14(3):1199-1211. doi:10.1002/jcsm.13225

8. Wang C, Schmid CH, Hibberd PL, et al. Tai Chi is effective in treating knee osteoarthritis: a randomized controlled trial. Arthritis Rheum. 2009;61(11):1545-1553. doi:10.1002/art.24832

9. Chenchen Wang, Christopher H. Schmid, Roger A. Fielding, et al. Effect of tai chi versus aerobic exercise for fibromyalgia: comparative effectiveness randomized controlled trial. BMJ. 2018;360:k851. doi:10.1136/bmj.k851

10. Wehner C, Blank C, Arvandi M, Wehner C, Schobersberger W. Effect of tai chi on muscle strength, physical endurance, postural balance and flexibility: A systematic review and meta-analysis. BMJ Open Sport Exerc Med. 2021;7(1):e000817. doi:10.1136/bmjsem-2020-000817

11. Zhang T, Mao M, Sun W, et al. Effects of a 16-week tai chi intervention on cutaneous sensitivity and proprioception among older adults with and without sensory loss. Res Sports Med Print. 2021;29(4):406-416. doi:10.1080/15438627.2021.1906673

12. Zhu GC, Chen KM, Belcastro F. Effects of different mind-body exercises in managing chronic pain of older adults: A network meta-analysis of randomized trials. Geriatr Nurs N Y N. 2024;55:183-190. doi:10.1016/j.gerinurse.2023.11.009

13 Qiao H, Hao X, Wang G. Effects of mind-body exercise on knee osteoarthritis: A systematic review and meta-analysis of randomized controlled trials. BMC Musculoskelet Disord. 2024;25(1):229. doi:10.1186/s12891-024-07278-4

14. Liang-Kung Chen, Jean Woo, Prasert Assantachai, et al. Asian Working Group for Sarcopenia: 2019 Consensus Update on Sarcopenia Diagnosis and Treatment. J Am Med Dir Assoc. 2020;21(3):300-307.e2. doi:10.1016/j.jamda.2019.12.012

15. J. H. Kellgren, J. S. Lawrence. Radiological assessment of osteo-arthrosis. Ann Rheum Dis. 1957;16(4):494-502. doi:10.1136/ard.16.4.494

16. Podsiadlo D, Richardson S. The timed “up & go”: A test of basic functional mobility for frail elderly persons. J Am Geriatr Soc. 1991;39(2):142-148. doi:10.1111/j.1532-5415.1991.tb01616.x

17. Shumway-Cook A, Brauer S, Woollacott M. Predicting the probability for falls in community-dwelling older adults using the timed up & go test. Phys Ther. 2000;80(9):896-903.

18. Mostafaee N, Pirayeh N, Fakoor M. Responsiveness and minimal clinically important changes of common patient-reported and performance-based outcome measures of physical function in patients with knee osteoarthritis. Physiother Theory Pract. 2024;40(11):2661-2669. doi:10.1080/09593985.2023.2269241

19. Angst F, Aeschlimann A, Stucki G. Smallest detectable and minimal clinically important differences of rehabilitation intervention with their implications for required sample sizes using WOMAC and SF-36 quality of life measurement instruments in patients with osteoarthritis of the lower extremities. Arthritis Rheum. 2001;45(4):384-391. doi:10.1002/1529-0131(200108)45:4%3C384::AID-ART352%3E3.0.CO;2-0

20. Kim MS, Koh IJ, Choi KY, et al. The minimal clinically important difference (MCID) for the WOMAC and factors related to achievement of the MCID after medial opening wedge high tibial osteotomy for knee osteoarthritis. Am J Sports Med. 2021;49(9):2406-2415. doi:10.1177/03635465211016853
