# Supplementary material for: Effect of 8-form Tai Chi vs health education on mobility in older adults with sarcopenic knee osteoarthritis: A randomized clinical trial
Source: Osteoarthr Cartil Open. 2026 Sep 23;8(4):100881. doi: 10.1016/j.ocarto.2026.100881 (PMC13675917; doi:10.1016/j.ocarto.2026.100881)
Supplement: Supplementary file 3 [file mmc3.docx]

**Statistical Analysis Plan (SAP)**

Eight-Form Tai Chi vs Health Education for Sarcopenic Knee Osteoarthritis (SPKOA): Randomized Clinical Trial

Version 1.2 | Date: 2024-12-20

**Sponsor/Institution:** Shanghai community-based trial (investigator-initiated)

**Planned sample size:** 96 participants (48 per group)

**Analysis software:** R (lme4/lmerTest/emmeans; mice)

# 1. Purpose and scope

This SAP prespecifies the statistical analyses for the parallel-group randomized clinical trial comparing a standardized 12-week 8-form Tai Chi program (TCG) with a health education control program (HEG) in older adults with sarcopenic knee osteoarthritis (SPKOA). The SAP aligns with CONSORT reporting guidance^35 and SPIRIT protocol standards^36

Unless otherwise specified, analyses are intention-to-treat (ITT) and use two-sided tests with α=0.05.

# 2. Study design summary

- Design: assessor-masked, 2-arm, individually randomized, superiority trial (1:1 allocation).
- Intervention period: weeks 0–12; follow-up through week 26.
- Assessments: baseline (week 0), weeks 4, 8, 12, and 26 for performance and patient-reported outcomes; ASMI at baseline and week 12 only.

# 3. Objectives, endpoints, and estimands

## 3.1 Primary objective and endpoint

Primary endpoint: Timed Up and Go (TUG) test time (seconds) at weeks 0, 4, 8, 12, and 26. Primary time point: week 12.

**Primary estimand (superiority):**

Adjusted between-group difference in change from baseline in TUG at week 12, expressed as (TCG − HEG). Negative values indicate greater improvement (larger reduction in TUG time) with Tai Chi.

## 3.2 Secondary objectives and endpoints

- Knee pain intensity: Knee Visual Analog Scale (KVAS; 0–100 mm).
- WOMAC total score (0–240) and subscales (pain, stiffness, physical function).
- Grip strength (kg).
- Modified Falls Efficacy Scale (MFES; 0–140).
- Appendicular skeletal muscle mass index (ASMI; kg/m²; baseline and week 12 only).

Secondary estimands mirror the primary estimand at weeks 12 and 26: adjusted between-group differences in change from baseline (TCG − HEG).

# 4. Analysis populations

- Intention-to-treat (ITT): all randomized participants, analyzed according to randomized group.
- Per-protocol (PP): participants meeting adherence thresholds (≥80% supervised sessions and ≥80% prescribed home-practice days) and with week 26 assessment; used for sensitivity analyses.
- Safety population: all randomized participants with any postrandomization safety information.

# 5. General statistical principles

- Two-sided α=0.05 for the primary endpoint at week 12.
- 95% confidence intervals (CIs) will be reported for effect estimates.
- Continuous outcomes will be summarized as mean (SD) at each time point by group.
- No formal multiplicity adjustment is planned for secondary outcomes; these are interpreted as supportive/exploratory.

# 6. Data handling and derivations

## 6.1 Baseline definition

Baseline is defined as the pre-randomization assessment at week 0 (TUG_1, KVAS_1, etc.).

## 6.2 Direction of benefit

For TUG, KVAS, and WOMAC outcomes, negative change indicates improvement. For grip strength, MFES, and ASMI, positive change indicates improvement.

## 6.3 Derived outcomes for reporting

Adjusted mean changes from baseline by group at each follow-up time point (weeks 4, 8, 12, 26) and adjusted between-group differences in change from baseline will be derived from the mixed-effects model via estimated marginal means (EMMs). Specifically, the between-group difference in change from baseline at week t is calculated as:

*DD(t) = [μ̂_TCG(t) − μ̂_TCG(0)] − [μ̂_HEG(t) − μ̂_HEG(0)]*

where μ̂_group(time) are the covariate-adjusted EMMs. DD(t) corresponds to the group×week interaction contrast, not the simple group difference at time t.

# 7. Primary analysis

The primary analysis will use a linear mixed-effects model (LMM) fitted by restricted maximum likelihood (REML) with participant-specific random intercepts.

**Model specification (TUG):** TUG_ij = β0 + β1·Group_i + β2·Week_j + β3·(Group_i×Week_j) + β4·Age_i + β5·Sex_i + β6·BMI_i + β7·KL_i + b0i + ε_ij.

Week is treated as a categorical factor (0, 4, 8, 12, 26). The primary estimand is DD(12) derived from EMMs as described in Section 6.3.

Covariates (prespecified): age (years), sex (male/female), body mass index (BMI; kg/m²), and Kellgren–Lawrence grade (I–IV).

## 7.1 Effect measure and reporting

Results will be reported as adjusted mean changes from baseline (95% CI) for each group and adjusted between-group differences in change from baseline (95% CI), with two-sided P values.

# 8. Secondary outcome analyses

Secondary continuous outcomes measured at weeks 0, 4, 8, 12, and 26 will be analyzed using the same LMM framework as the primary endpoint. ASMI (baseline and week 12 only) will be analyzed with an LMM including the two time points (week factor: 0 and 12).

# 9. Missing data

## 9.1 Extent and patterns

Missingness will be summarized by group and visit. Baseline characteristics will be compared between participants with and without observed TUG at week 12 and week 26 using descriptive statistics and two-sample t tests.

## 9.2 Primary assumption

The primary LMM uses maximum likelihood–based estimation (REML) under a missing-at-random (MAR) assumption conditional on observed outcomes and covariates.

## 9.3 Sensitivity to departures from MAR (MNAR)

To assess robustness to plausible departures from MAR, a pattern-mixture sensitivity analysis will be conducted using delta-adjusted multiple imputation. For participants with missing follow-up TUG, imputed values will be shifted by prespecified deltas (e.g., +0.5 s, +1.0 s, +1.5 s) in both groups, and additionally under differential deltas by group (eg, larger penalty in the intervention group) to create tipping-point scenarios.

# 10. Prespecified sensitivity analyses

1. Alternative random-effects structure: linear-time model with centered time and participant-specific random slope (time_c || participant), compared with the primary week-factor random-intercept model.
2. Complete-case analyses restricting the sample to participants with observed TUG at week 12 and, separately, those with observed TUG at week 26; refit the primary model.
3. Outlier-removed analysis excluding observations with |standardized residual| > 3 from the primary model; refit the primary model.
4. Multiple imputation (MI): two-level joint modeling using mice method 2l.pan in long format with participant as the clustering variable; 20 imputations; DD contrasts pooled using Rubin’s rules.
5. Per-protocol analysis in the PP population using the primary week-factor model.

# 11. Clinically important improvement and responder analyses

Responder analyses were prespecified using minimal clinically important difference (MCID) thresholds: TUG improvement ≥2.8 seconds; KVAS reduction ≥15 mm; WOMAC total score reduction ≥40 points. Responder status at weeks 12 and 26 will be defined as baseline − follow-up ≥ threshold. Analyses will follow the intention-to-treat principle with denominators including all randomized participants (N=48 per group); participants with missing follow-up data will be conservatively classified as nonresponders. For each outcome and time point, we will report No./N (%), risk differences (TCG − HEG, percentage points) and risk ratios with 95% CIs. Two-sided P values for between-group differences in responder proportions will be calculated using χ² tests or Fisher exact tests, as appropriate; these tests are exploratory.

# 12. Subgroup analyses

A priori subgroup analyses will evaluate effect modification on the primary estimand by including subgroup×group×week interaction terms. Subgroups include:

- Sex (male vs female).
- Age (<75 vs ≥75 years).
- BMI (<24 vs ≥24 kg/m²).
- KL grade (I–II vs III–IV).

Subgroup analyses are exploratory and will be interpreted cautiously.

# 13. Safety analyses

Adverse events (AEs) will be collected throughout the intervention and follow-up using structured queries at study visits and participant self-report. Serious adverse events (SAEs) will be defined as events resulting in hospitalization, disability, or death. AEs will be coded using a standard medical dictionary (eg, MedDRA) and graded by severity (mild, moderate, severe) when applicable; relatedness to study participation will be assessed by investigators (possibly related vs unrelated/unlikely related). Safety will be reported in two tiers: the main manuscript will present a concise summary of participants with ≥1 AE, ≥1 SAE, AEs possibly related to study participation, and withdrawals due to AEs (Table 4); the Supplement will provide a detailed listing by system organ class/preferred term, severity, relatedness, and time window (weeks 0–12 and weeks 12–26) (eTable 3).

# 14. Software and reproducibility

Analyses will be conducted using R (R Foundation for Statistical Computing). Key packages include lme4/lmerTest for mixed-effects models, emmeans for marginal means and contrasts, mice for multiple imputation, and ggplot2 (with ragg/cairo where needed) for figures.

Code will be version-controlled and executed on the locked analysis dataset. Any deviations from this SAP will be documented with rationale and dated prior to unblinding of the final database.
